# Documenting Biogeographical Patterns of African Timber Species Using Herbarium Records: A Conservation Perspective Based on Native Trees from Angola

**DOI:** 10.1371/journal.pone.0103403

**Published:** 2014-07-25

**Authors:** Maria M. Romeiras, Rui Figueira, Maria Cristina Duarte, Pedro Beja, Iain Darbyshire

**Affiliations:** 1 Tropical Botanical Garden, Tropical Research Institute (IICT), Lisbon, Portugal; 2 Centre for Biodiversity, Functional and Integrative Genomics (BIOFIG), Faculty of Sciences, University of Lisbon, Lisbon, Portugal; 3 CIBIO - Research Center in Biodiversity and Genetic Resources/InBIO, University of Porto, Vairão, Portugal; 4 Royal Botanic Gardens, Kew. Richmond, United Kingdom; CNR, Italy

## Abstract

In many tropical regions the development of informed conservation strategies is hindered by a dearth of biodiversity information. Biological collections can help to overcome this problem, by providing baseline information to guide research and conservation efforts. This study focuses on the timber trees of Angola, combining herbarium (2670 records) and bibliographic data to identify the main timber species, document biogeographic patterns and identify conservation priorities. The study recognized 18 key species, most of which are threatened or near-threatened globally, or lack formal conservation assessments. Biogeographical analysis reveals three groups of species associated with the enclave of Cabinda and northwest Angola, which occur primarily in Guineo-Congolian rainforests, and evergreen forests and woodlands. The fourth group is widespread across the country, and is mostly associated with dry forests. There is little correspondence between the spatial pattern of species groups and the ecoregions adopted by WWF, suggesting that these may not provide an adequate basis for conservation planning for Angolan timber trees. Eight of the species evaluated should be given high conservation priority since they are of global conservation concern, they have very restricted distributions in Angola, their historical collection localities are largely outside protected areas and they may be under increasing logging pressure. High conservation priority was also attributed to another three species that have a large proportion of their global range concentrated in Angola and that occur in dry forests where deforestation rates are high. Our results suggest that timber tree species in Angola may be under increasing risk, thus calling for efforts to promote their conservation and sustainable exploitation. The study also highlights the importance of studying historic herbarium collections in poorly explored regions of the tropics, though new field surveys remain a priority to update historical information.

## Introduction

Legacy data from natural history collections contain invaluable information about biodiversity in the recent past, providing a baseline for detecting change and forecasting future trends [1]. In the case of plants, specimens have accumulated for hundreds of years in herbaria, and these may be used as the basis for identifying threatened or declining species, guiding future research and monitoring programs, and establishing conservation priorities [2]. For instance, the IUCN Sampled Red List Index for plants was driven in its first iteration almost solely by herbarium specimen data [3]. Data from herbaria are particularly important in poorly explored regions of the tropics, where the lack of continuous field-based botanical research has emphasized the pivotal role of herbaria in documenting plant diversity and species distributions [4]–[6]. The interest in herbaria for undertaking conservation biology research has thus grown in recent years, though less than about 2% of the herbarium specimens have been used to answer biogeographical or environmental questions [6].

Establishing baselines is particularly important for those tropical tree species that are exploited commercially and have come under increasing pressure from the global timber trade [7]–[8]. Over-exploitation has resulted in declining populations of the most valuable timber species and it is one of the foremost causes for the loss and degradation of tropical forests [9], with utmost negative consequences for the conservation of biodiversity and ecosystem services [10]–[11]. In recent decades, efforts have been made to increase the sustainability of tropical timber exploitation, through for instance the outright ban on or severe restrictions to the trade of endangered species, or the implementation of certification schemes for timber harvested sustainably [8]–[12]. These approaches face several problems, however, including uncertainties related to the conservation status of many exploited species due to insufficient knowledge of their distribution, abundance and population trends [13]–[15]. Although this type of information has become increasingly available for tropical forests of Central and South America [16]–[17] and Asia [18]–[19], data are still very limited for most African forests [20]. Considering that Africa still holds some of the most important tropical forests in the world [21]–[22] and that these have been increasingly exploited [23], information on the conservation status of its timber species is urgently required [24].

Angola is one of the African countries for which basic data on timber tree species are most severely lacking, though the country has a forested area of about 40–60 million hectares largely administered by the government [25]–[26]. Deforestation rates in Angola are among the highest in Sub-Saharan Africa [27], which is likely a consequence of wood extraction for firewood and charcoal, slash-and-burn cultivation, urban expansion, and logging [25]. Illegal logging of valuable timber is considered one of the potential causes of forest degradation, but there is no information on the extent of this problem [25]. Despite some early studies [28]–[32], botanical data on the forests of Angola are scarce because most of the country was inaccessible to researchers during the war of independence (1961–1974), and the subsequent civil war (1975–2002). Despite increases in safety during the first decade of the 21st century, field biodiversity research has remained very limited, thereby making historic herbarium specimens the main source of data for studying the distribution patterns of tree species exploited commercially in Angola. This information is urgently required because Angola is currently experiencing rapid economic and human population growth, which is likely to place further pressure on its forest resources, with negative consequences for biodiversity, ecosystem services, and ultimately for human well-being [25]. Data on timber trees is also required to inform ongoing initiatives to improve the protected area network of Angola [33].

The present study focuses on the timber trees of Angola, combining herbarium and bibliographic data to assess biogeographical patterns and conservation priorities, thereby providing baseline information required for their conservation management and sustainable exploitation. Specifically, the study aims (i) to inventory the timber tree species of Angola based on a thorough review of literature and data held in herbaria, (ii) to document biogeographical patterns of the timber species in relation to WWF ecoregions [34]; and (iii) to estimate species conservation priorities based on distribution patterns, representation in protected areas and deforestation rates.

## Materials and Methods

### Study area

**Figure 1:**
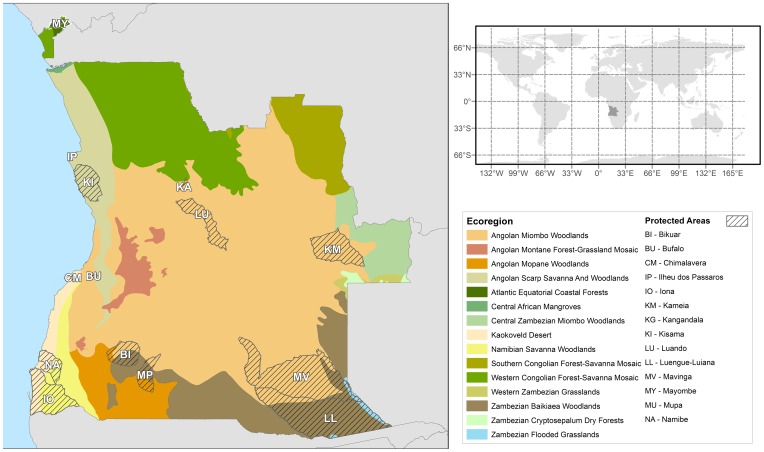
Map of Angola. The 15 WWF ecoregions represented in Angola are displayed together with the network of protected areas (see text for details).

The Republic of Angola (Fig. 1) is the largest country in southern Africa (1.24 million km^2^), encompassing a variety of climatic characteristics, which correspond to five climate types by the Köppen–Geiger system [35]. The phytogeographic study of Grandvaux-Barbosa [36] identified 32 vegetation units in the country, ranging from rainforests in the northwest to the desert in the southwest. The global ecoregions map of World Wildlife Fund (WWF) [34] recognises the presence of 15 biogeographic units in Angola (Fig. 1), of which the most widespread are the miombo woodlands of the central plateau, and the western Congolian forest-savanna mosaics in the north. Other important but less widespread forest types include the Atlantic Equatorial coastal forests in Cabinda, the mopane (*Colophospermum mopane*) woodlands, and the Namibian savanna woodlands in the southwest (see Fig. 1). The network of protected areas was mainly established in colonial times to protect large ungulates, and it has been considered too limited to adequately protect most biodiversity components, notably vascular plants [25], [33].

### Species data

Data on the timber species of Angola were obtained through a combination of bibliographic sources and the study of 2670 herbarium records: 417 of Angolan specimens (see Table S1) and 2253 records from 62 providers available through the GBIF data portal (Tables S2 and S3). First, a thorough literature review was undertaken, focusing on studies of the flora of Angola [36]–[44], and on studies documenting the use of afro-tropical timber trees [28]–[32]. Based on this information, we selected for further analysis the subset of timber trees that are: (i) known to be native in Angola; (ii) documented in the Angolan literature to be exploited for timber in colonial times or at present; and (iii) traded in international timber markets. Many of these timber species are important components of the upper forest layer, above 25 m, and all are known for their economic value, thus making them interesting from both ecological and conservation standpoints. For each species selected, we compiled information on their distribution in Angola and across Africa, their habitat, ecology, timber value and characteristics, and their global conservation status based on the IUCN Red List of Threatened Species [45].

Second, a thorough study of herbarium specimen data was undertaken for all timber species selected. The research was concentrated on herbaria holding the largest collections of Angola vascular plants, including LISC (Tropical Research Institute), LISU (University of Lisbon), COI (University of Coimbra), BM (Natural History Museum, London), and K (Royal Botanic Gardens Kew). The collecting locality for each specimen was georeferenced wherever possible, using 1∶100,000 cartographic maps and geographic gazetteers [46], and data was compiled in a geographic database prepared in ArcGIS Arcinfo ver. 10.0 [47]. Further information about the global native distribution of each selected timber species in Africa was gathered from the GBIF data portal. Although it is recognised that GBIF does not contain all known records of the species studies, it is deemed adequate to provide a first approximation of their geographic range.

### Biogeographic patterns

Patterns of timber tree species distribution in Angola were analysed in relation to the 15 WWF ecoregions identified in the country [34]. We focused on WWF ecoregions because they have been produced mainly as a utility tool for conservation planning [48], and so it was considered important to examine whether they could be used as meaningful spatial units for conservation prioritization and management of Angolan timber tree species.

Analyses were based on a presence/absence matrix, which indicated whether or not each timber tree species had been recorded within each WWF ecoregion. Presence/absence was used instead of the number of records, to reduce the bias associated with geographic variation in sampling effort. Although this approach does not avoid the problem of false absences (i.e. absence due to lack of sampling rather than a true record of absence), we believe that this problem has been minimised by using a small number of spatial units, each covering a large geographic area and encompassing many species records. Hierarchical clustering was then carried out, using the Jaccard index as a measure of similarity between species distribution, and the Ward agglomerative procedure [49]. The Jaccard index was used because it does not consider double absences [49]. Several agglomerative methods were tested (e.g., UPGMA, WPGMA), but they produced largely similar results. Clusters identified at different levels of the dendrogram were mapped and checked for spatial consistency, i.e., whether each group was associated with a well-defined spatial region, and we selected the number of clusters that maximised spatial interpretability [49]. Quantitative approaches such as the L-Method [50] were also tested but the number of clusters produced was excessively large and with no spatial consistency. Analyses were carried out using ‘dist’ and ‘hclust’ functions implemented in R version 3.02 [51].

The spatial distribution of the species groups emerging from the cluster analysis was overlapped with the WWF ecoregions map, and spatial consistency between species groups and ecoregions was visually inspected. A similar investigation was carried out by overlapping the spatial distribution of species groups and the climate classification map of Köppen–Geiger [35].

### Species conservation priorities

Estimating conservation priorities from herbaria data is difficult, because a species may no longer exist in localities where it was historically recorded, and because collectors may be biased towards or against certain species or regions [2], [5]. To overcome these problems, we used a combination of three relatively coarse criteria, which were judged useful in helping to guide future conservation efforts, despite some potential shortcomings and limitations.

A first approximation for conservation prioritization was obtained by computing the extent of occurrence (EOO) of each species, assuming that the highest priority should be given to species with a small EOO in Angola, and to species with a large proportion of its global EOO concentrated in the country. EOO was computed from the georeferenced locality data for each species, using the minimum convex hull polygon method [52], implemented in GEOCAT [53]. Computations were carried out at the scale of the African Continent and that of Angola, and we calculated Angola's contribution to the overall EOO for each species. Areas offshore from the African continent were calculated using ArcGIS Arcinfo ver. 10.0 [47] and were excluded from the EOO polygon. Although the area of occupancy (AOO) is an important parameter to assess species conservation status [52], it was not estimated because large gaps in species distribution are likely to be due primarily to the lack of comprehensive field surveys or lack of data reporting by herbaria to GBIF, rather than resulting from true species absences.

A second indicator of conservation priority was based on the occurrence of herbarium specimens' locations in national parks and reserves, assuming that a higher conservation risk should be attributed to the species poorly represented within protected areas. We considered both the number of locations recorded within protected areas, and the percentage of the EOO that is included in protected areas. Although we recognise that it is uncertain whether a given species occurs at any particular location within its EOO, we assumed that the overlap between EOO and protected areas could be taken as a coarse approximation of the relative representation of a species within the protected area network. The geographical limits of protected areas were obtained in GIS shape file format from WDPA [54]. New protected areas unavailable in WDPA were digitised in ArcGIS ArcInfo ver. 10.0 [37] from maps published in the official journal of The Republic of Angola (law n° 38/11 of December, 29 2011, p. 6340).

Finally, conservation priority was also evaluated by estimating rates of forest loss between 2000 and 2012 around the georeferenced localities for each species. We assumed that higher conservation priority should be given to species occurring in areas with low forest cover, and where the recent deforestation rate is highest. Forest cover was estimated for each georeferenced specimens location using raster maps provided by Hansen et al. [27], by multiplying the percent tree (crown) cover per pixel and the pixel area (30-m resolution), and then summing across all pixels extracted in a 5-km buffer of the location. Deforestation rate was calculated by estimating the area of pixels showing forest loss, and then expressing it as a percentage of total tree cover in 2000. Similar analyses were carried out using 1, 2.5 and 10-km buffers, but the results were much the same, and so they were not considered further.

## Results

From the literature review and the study of herbarium specimens, we identified eighteen native timber species occurring in Angola (Table 1), which have a high commercial value due to the quality of their timber (Table S4). Available herbarium data are rather old, corresponding primarily to specimens collected in 1850–1860, 1910–1920, and 1950–1975 (Fig. S1). Most species (>80%) belong to the Fabaceae and Meliaceae families, and they are associated with tropical rainforests (11 species), evergreen forests and woodlands (2 species), and mainly with dry forests and savannas (5 species) (Table 1, Table S4). Half the species are either classified as threatened (7) or near-threatened (2) by IUCN at the global scale, whereas the conservation status of eight species has not yet been evaluated.

**Table 1:** Geographical distribution in Angola and in the African continent, main types of ecosystems and global conservation status [45] for each species considered in the present study. Nomenclature according to The Plant List [55].

Main types of ecosystems	Geographical distribution		Conservation status and criteria
Species	Angola (district)	Africa	
***Tropical Rainforests***			
*Bobgunnia fistuloides* (Harms) J.H. Kirkbr. & Wiersema	Cabinda; Malanje; Zaire	Nigeria, Cameroon, Gabon, Angola, D.R. Congo, Mozambique.	Least Concern
*Entandrophragma angolense* (Welw.) C. DC.	Cabinda; Cuanza Norte; Cuanza Sul; Malanje	From Guinea to Uganda, Kenya and Angola	Vulnerable A1cd
*Entandrophragma candollei* Harms	Cabinda	From the Ivory Coast to Angola and D.R. Congo	Vulnerable A1cd
*Entandrophragma cylindricum* (Sprague) Sprague	Cabinda	From Sierra Leone to Cabinda and Uganda	Vulnerable A1cd
*Entandrophragma utile* (Dawe & Sprague) Sprague	Cabinda	From the Ivory Coast to Angola, D.R. Congo and Uganda	Vulnerable A1cd
*Gossweilerodendron balsamiferum* (Vermoesen) Harms	Cabinda	From the south of Nigeria and Cameroon to D.R. Congo and Angola	Endangered A1cd
*Guibourtia arnoldiana* (De Wild. & T. Durand) J. Léonard	Cabinda; Zaire	Gabon, Congo, Angola (Cabinda), D. R. Congo (Maiombe)	Not Evaluated
*Khaya ivorensis* A. Chev.	Cabinda	From the Ivory Coast to Angola (Cabinda)	Vulnerable A1cd
*Milicia excelsa* (Welw.) C.C. Berg	Cabinda; Cuanza Norte	Widely distributed in Africa, from Senegal to Angola, D.R. Congo, East Africa and Mozambique	Near Threatened
*Oxystigma oxyphyllum* (Harms) J. Léonard	Cabinda	From Nigeria to Angola (Cabinda)	Not Evaluated
*Terminalia superba* Engl. & Diels	Cabinda	From Guinea to D.R. Congo (Maiombe)	Not Evaluated
***Evergreen Forests and Woodlands***			
*Khaya anthotheca* (Welw.) C. DC.	Bengo; Cuanza Norte; Malanje	From Sierra Leone to Uganda and Tanzania, and central Angola, Zambia, Malawi, Mozambique and Zimbabwe; also in the Ivory Coast, the Gold Coast, Nigeria and Cameroon.	Vulnerable A1cd
*Pterocarpus tinctorius* Welw.	Bengo; Cuanza Norte; Cuanza Sul; Luanda; Malanje; Zaire	Congo, Angola, Zambia, Zimbabwe, Tanzania and Mozambique	Not Evaluated
***Dry Forests, Woodlands and Savannas***			
*Afzelia quanzensis* Welw.	Benguela; Bie; Cuando Cubango; Cuanza Norte; Cuanza Sul; Cunene; Huila; Malanje; Namibe	In Angola, Namibia, D.R. Congo, Zambia, Zimbabwe and Botswana, and from Somalia to South Africa	Not Evaluated
*Diospyros mespiliformis* Hochst. ex A. DC.	Bengo; Cuando Cubango; Cuanza Norte; Cuanza Sul; Cunene; Huila; Luanda; Namibe	From Senegal to Sudan, and southwards to Namibia. It can be found from the mouth of the Zaire river to the Transvaal and South Mozambique, but not in the Guineo-Congolian rainforests	Not Evaluated
*Entandrophragma spicatum* (C. DC.) Sprague	Benguela; Cunene; Huila; Namibe	Southern Angola and Namibia	Not Evaluated
*Guibourtia coleosperma* (Benth.) J. Léonard	Bengo; Bie; Cuando Cubango; Cunene; Huambo; Huila; Lunda Norte; Lunda Sul; Moxico	D.R. Congo, Angola, Namibia, Botswana, Zambia, Zimbabwe	Not Evaluated
*Pterocarpus angolensis* DC.	Benguela; Bie; Cuando Cubango; Cuanza Norte; Cuanza Sul; Cunene; Huambo; Huila; Lunda Norte; Lunda Sul; Malanje; Moxico; Namibe; Uige	From Congo to Namibia and from Tanzania to Swaziland	Near Threatened

**Figure 2:**
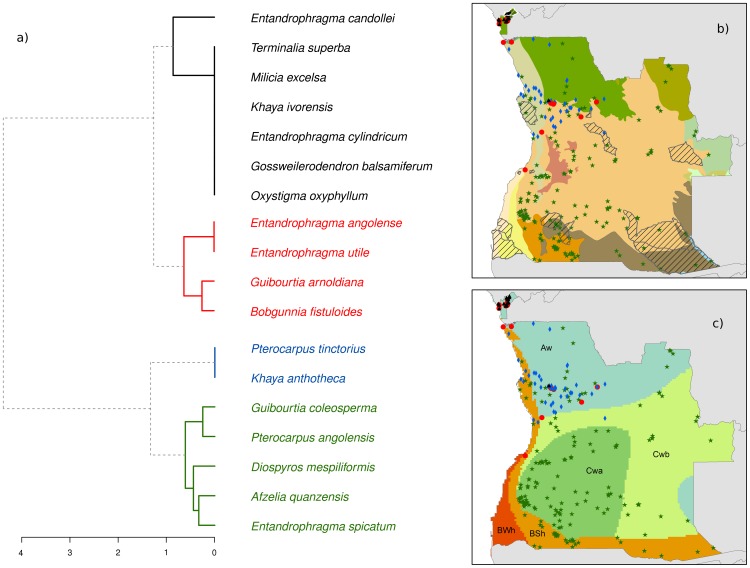
Biogeographical patterns of Angolan forest species selected for this study. **a**) Dendrogram of a cluster analysis based on the distribution (presence/absence) of timber tree species in each of the ecoregions. Distribution of collection localities of the four species groups identified in this study, in relation to: **b**) protected areas and the 15 WWF ecoregions represented in Angola; and **c**) Köppen–Geiger climate classification. Clustering was based on the Jaccard index of similarity and on the Ward agglomeration algorithm.

**Figure 3:**
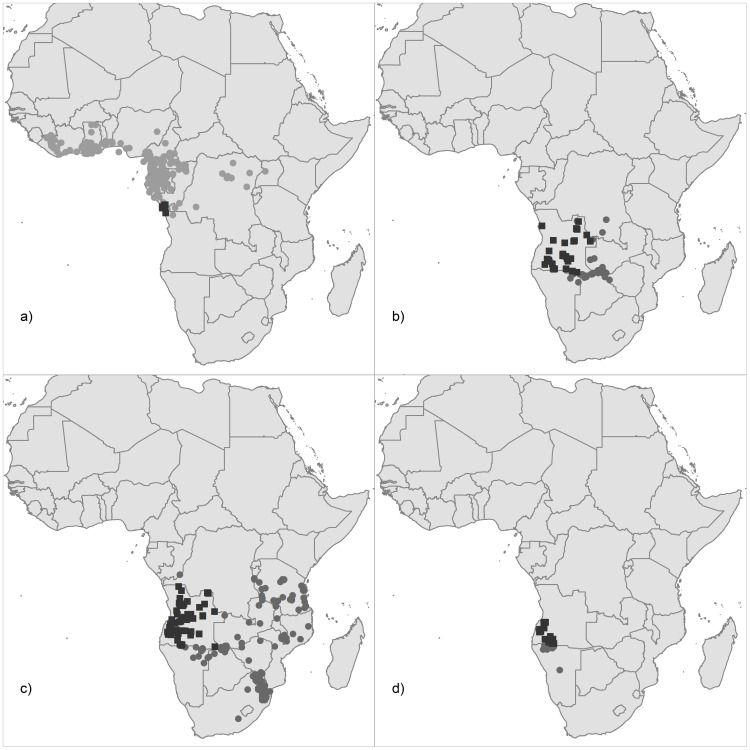
Geographic distribution in Africa of representative timber trees from Angola. (**a**) Species typical of Guineo-Congolian rainforests that reach their southern limit in Cabinda (*E. cylindricum; E. utile; G. arnoldiana; G. balsamiferum; K. ivorensis; O. oxyphyllum; T. superba*); and species with >15% of their global range concentrated in Angola, including (**b**) *G. coleosperma*, (**c**) *P. angolensis*, (**d**) *E. spicatum*. Black squares: studied specimens from Angola, housed in LISC, LISU, COI, BM and K; grey circles: data obtained via the GBIF portal.

The cluster analysis of timber trees identified four groups of species (Fig. 2a). The first and second groups have similar spatial patterns, with most occurrences concentrated in the small enclave of Cabinda. The second group, however, is also represented in north-western regions of Angola. For eight of the eleven species in these two groups, Cabinda is the southern limit of wider distributions concentrated in the Guineo-Congolian rainforests (Fig. 3a). The third group includes only two species and it has a distribution concentrated in north-western regions of Angola, though it is absent from Cabinda. The fourth group includes five species, and it occupies most of the Angolan territory, with the exception of Cabinda.

There is a poor match between the spatial distribution of the four species groups and the WWF ecoregions, as each group occurs in several ecoregions (Fig. 2b). Overlay with the climate classification map of Köppen–Geiger suggests a rough association between groups 1–3 and a single climate type (Aw - Equatorial savanna with dry winter), while group 4 occurs in a wide range of climate types (Fig. 2c).

Most of the timber species have a large global extent of occurrence - EOO (Table 2). Smaller EOO values are found for *Entandrophragma spicatum* and *Guibourtia arnoldiana* (≈1×10^5^ km^2^), but they are still one order of magnitude above the threshold for species qualifying as threatened under IUCN criterion B (i.e., >2×10^4^ km^2^) (Table 2). Within Angola, however, there are nine species with a restricted EOO (<2×10^4^ km^2^) and thus potentially qualifying as threatened at the national level. Three species have more than 15% of their global EOO concentrated in Angola (Fig. 3b–d), reaching >50% in the case of *E. spicatum* and *G. coleosperma* (Table 2). Few of the historical herbarium specimens were collected from within current protected areas, with ≤5 localities for all species evaluated (Table 2). More than 10% of the EOO of eight species overlaps with protected areas, whereas there was no overlap for another six species (Table 2).

**Table 2:** Number of specimen collection localities and estimates of the global and national (Angola) extent of occurrence (EOO) for the selected timber tree species.

Species	Number of localities			Extent of Occurrence			%EOO	%EOO protected
	Global	Angola	Protected areas	Global (×10^6^ km^2^)	Angola (×10^3^ km^2^)	Protected areas (×10^3^ km^2^)		
*Afzelia quanzensis*	107	34	3	5.2	429.6	56.7	8.3	13.2
*Bobgunnia fistuloides*	47	5	1	0.8	0.2	0.0	0.0	0.0
*Diospyros mespiliformis*	272	37	3	12.4	464.5	96.5	3.8	20.8
*Entandrophragma angolense*	67	8	1	2.7	169.6	0.5	6.2	0.3
*Entandrophragma candollei*	42	1	1	1.6	a	a	a	a
*Entandrophragma cylindricum*	50	3	1	1.9	0.01	0.0	0.0	0.0
*Entandrophragma spicatum*	13	21	1	0.1	75.1	12.3	52.5	16.4
*Entandrophragma utile*	46	2	1	1.6	a	a	a	a
*Gossweilerodendron balsamiferum*	30	9	3	1.0	1.4	0.1	0.1	10.5
*Guibourtia arnoldiana*	10	5	0	0.1	10.1	0.0	11.2	0.0
*Guibourtia coleosperma*	36	32	5	1.3	697.0	89.6	55.4	12.9
*Khaya anthotheca*	76	8	0	6.0	58.4	0.0	1.0	0.0
*Khaya ivorensis*	49	5	0	1.0	0.6	0.0	0.1	0.0
*Milicia excelsa*	156	3	1	7.1	0.1	0.0	0.0	0.0
*Oxystigma oxyphyllum*	34	6	2	1.3	1.1	0.3	0.1	28.1
*Pterocarpus angolensis*	135	61	2	4.8	856.2	113.3	17.7	13.2
*Pterocarpus tinctorius*	45	38	1	2.1	158.6	10.3	7.5	6.5
*Terminalia superba*	98	6	2	2.0	0.7	0.1	0.0	14.3

% EOO is the percentage contribution of Angola to the global extent of occurrence; % EOO protected is the percentage of the EOO within Angola which is included in protected areas network.

a EOO not estimated due to insufficient data (≤2 locations).

Forest cover, in 2000, around the location of collection localities ranged from <20% in the case of *Diospyros mespiliformis*, *E. spicatum* and *G. coleosperma*, to >70% in the case of *Entandrophragma candollei*, *Milicia excelsa*, *Oxystigma oxyphyllum* and *Terminalia superba* (Table 3). Variation among species in deforestation rate (2000–2012) was less marked, but particularly high values (>10%) were recorded in the occurrence areas of *G. coleosperma* and *Pterocarpus angolensis*.

**Table 3:** Forest cover (in 2000) and forest cover changes (2000–2012) estimated in 5-km buffers around the herbarium collection localities for each Angolan timber tree species.

Species	N	Tree Cover (%)	Forest Gain (%)	Forest Loss (%)	Deforested (%)
*Afzelia quanzensis*	6	23.8	0.1	2.3	9.6
*Bobgunnia fistuloides*	8	60.6	0.8	4.5	7.4
*Diospyros mespiliformis*	18	13.6	0.1	0.6	4.6
*Entandrophragma angolense*	9	62.1	0.9	5.5	8.9
*Entandrophragma candollei*	13	77.2	1.6	6.6	8.6
*Entandrophragma cylindricum*	10	68.6	1.4	3.8	5.6
*Entandrophragma spicatum*	11	9.3	0.0	0.8	8.2
*Entandrophragma utile*	12	46.7	1.1	3.5	7.6
*Gossweilerodendron balsamiferum*	1	69.0	0.6	2.5	3.6
*Guibourtia arnoldiana*	7	55.9	0.8	1.5	2.8
*Guibourtia coleosperma*	3	18.9	0.0	5.1	27.1
*Khaya anthoteca*	14	52.8	0.4	4.9	9.2
*Khaya ivorensis*	15	48.8	0.5	1.7	3.6
*Milicia excelsa*	16	84.4	0.9	4.2	5.0
*Oxystigma oxyphyllum*	2	76.8	0.7	2.8	3.6
*Pterocarpus angolensis*	4	20.4	0.0	3.0	14.7
*Pterocarpus tinctorius*	5	42.1	0.2	3.3	7.8
*Terminalia superba*	17	72.8	0.3	1.6	2.2

Tree cover, forest gain and forest loss are percentages expressed in relation to total buffer area. Deforestation rate is computed as the percentage of forest loss in 2012, in relation to total tree cover in 2000. Estimates were based on data extracted from Hansen et al. [27].

## Discussion

This study recognized the presence of 18 key timber tree species in Angola, most of which are widely used as timber trees elsewhere in Africa [26]. These species are important components of woody vegetation communities and are known for their economic value, thus making them important from both ecological and economic perspectives. Several of these species are highly valued in international timber markets and they have been historically exploited in Angola, including the African mahoganies (*Entandrophragma* spp.), the agba (*G. balsamiferum*), and the tchitola (*O. oxyphyllum*) [43], hence they are under increasing pressure in the country.

### Biogeographical patterns

This study revealed striking differences in biogeographic patterns of timber species in Angola, recognizing different groups associated with regions with relatively homogeneous climatic conditions (i.e. tropical rainforests; evergreen forests and woodlands; and dry forests, woodlands and savannas). Three of the four clusters identified were associated with Cabinda and the north-western regions of Angola, showing a close matching with the Aw climate category of Köppen–Geiger and with the Congolian region identified in the recent bioregionalization study of Linder et al. [56]. The timber species included in the first two clusters (ca. 60%) corresponding largely to Guineo-Congolian rainforest species [57], where the rainy season lasts for six months or more, and the relative air humidity is above 80%, with some areas having persistent and dense fogs (locally known as *cacimbo*). In Angola they occur in Cabinda's Maiombe forest (extending through Congo, Democratic Republic of the Congo and Angola), which are dense moist forest formations, with high ecological and floristic diversity [58], [59]. Most of the species found in Guineo-Congolian rainforests have their south-western range limit in Cabinda (see Fig. 3a), where they may face drier climatic conditions than within their core range. These peripheral populations may have unique adaptations to specific environmental conditions that are absent from other populations [60], and so may be particularly valuable in a warming scenario due to climate change [61].

The third cluster included just two species (*K. anthotheca* and *P. tinctorius*), and it was restricted to woodlands and evergreen forests of northwest Angola. This region is characterized by a rainy season lasting about six months and relative air humidity of about 75% [36]. Finally, the fourth cluster comprised five species and is widespread throughout the country, albeit little represented in the north-western regions of Angola. Species included in this group occur mainly in dry forests and savannas, and sometimes their distribution reach the semi-arid and arid regions of southern Angola, characterized by xerophytic vegetation. Among the studied timber species, the legumes *G. coleosperma* (see Fig. 3b) and *P. angolensis* (see Fig. 3c) are the most widespread in Angola, and are mainly found in miombo woodlands, which is the dominant forest component of Angola and one of the major dry forest-savanna biomes of the world [34].

The spatial distribution of the four clusters retrieved from the biogeographical analysis showed little concordance with the WWF ecoregions. The mismatch was particularly notable in the case of the Cabinda forests, which have a very unique set of timber tree species that are not adequately captured by WWF ecoregions. In fact, although one of the two regions dominating the enclave of Cabinda also occurs in a larger area in the northwest of Angola (western Congolian forest-savanna mosaic), most timber species characteristic of the former region were not found elsewhere. Reasons for the mismatches are uncertain, but they are probably related to the operation of climatic and historical factors that are not adequately captured by the WWF ecoregion definitions. Irrespective of the reason, however, these results suggest that WWF ecoregions may not provide an adequate operational basis for conservation planning exercises targeting timber tree species in Angola.

It is suggested that the biogeographic patterns observed in this study, might be better explained by the climatic classification of Köppen–Geiger [35], which suggests that the native range of these timber species is conditioned by large scale patterns. These regions are broadly similar to those recently proposed by Linder et al. [56], that demonstrate the existence of only seven well-defined and consistent biogeographical regions in sub-Saharan Africa, proposing that the best approach might be to recognize, as White [57] did, a small number of very broad biogeographical regions in Africa that can reflect the patterns found in both vertebrates and plants [56].

### Species conservation priorities

Our results suggest that at least 11 of the species evaluated should be given high conservation priority in Angola. These include: (1) globally threatened or near-threatened species with small ranges in Angola and largely restricted to Cabinda or, to a much lesser extent, the north-western regions (*E. angolense*, *E. candollei*, *E. cylindricum*, *E. utile*, *G. balsamiferum*, *K. anthotheca*, *K. ivorensis* and *M. excelsa*); and (2) species from dry forests with a large proportion of their global range concentrated in Angola, and occurring in areas that are affected at present by high deforestation rates (*E. spicatum*, *G. coleosperma* and *P. angolensis*).

From the first group, all but two species (*E. angolense* and *K. anthoteca*) are concentrated in Cabinda's Maiombe forest, with a very small EOO in Angola, though they are widely distributed elsewhere in Guineo-Congolian rainforests. The herbarium collections studied of all these species were made in locations still retaining a relatively extensive forest cover (46.7 to 84.4%), and where deforestation rates between 2000 and 2012 were lower than in other areas of Angola. The overlap between the EOO and protected areas for these species is negligible (<0.5%), except in the case of *G. balsamiferum* (10.5%). These species may thus remain largely unprotected in Angola despite the recent creation of the Maiombe National Park, which was specifically designed to protect the Cabinda's forest. This is worrying in view of ongoing logging activities in Cabinda, where these species may be under increasing pressure [25], [58].

The second group of conservation priority species includes timber trees that are mainly found in dry forests, and that are particularly important from a conservation perspective because their Angolan range represents a large proportion (17.7–55.4%) of their global range. Although these species have a relatively large overlap between their EOO and protected areas in Angola (12.9–16.4%), they occur in areas where tree cover is among the lowest for timber trees in Angola (9.3–20.4%). Further, the collection localities of these species have suffered high recent deforestation rates, amounting to >10% in 12 years for *G. coleosperma* and *P. angolensis*. These species should therefore merit national conservation attention, though only *P. angolensis* has been considered near-threatened in IUCN global assessment [45], while the evaluation of *E. spicatum* and *G. coleosperma* is lacking.

The conservation priorities identified in this study are limited because herbarium data may not reflect current distribution, the ecological information for most species is scarce and there are virtually no data on present logging pressure. Notwithstanding, we believe that our approach provides a first approximation for timber tree species prioritization in Angola, which may be a useful guide to conservation decisions until more detailed information becomes available.

### Conservation implications

Our study clearly underlines the need to take urgent action to protect the Cabinda's Maiombe forest, where there is a significant concentration of threatened timber species of high conservation priority. At present, these forests may be better conserved than similar Guineo-Congolian forests in neighbouring countries (see Fig. S2 in Supporting Information) where deforestation rates are high and concessions for industrial logging are expanding [27], [62]. However, the Cabinda's forest may be under increasing legal and illegal logging pressure, though data to quantify this problem are scarce [25]. According to Buza et al. [58], Cabinda is the largest producer of timber from Angola, being responsible for 33.9% of total timber exportations between 1990 and 1995; from 1996 to 2000 the external market consumed 85% of the logged timber. An important step towards the conservation of these forests has been the recent creation of the Maiombe National Park in a trans-frontier conservation area, as a result of an international cooperation between Angola, Congo and the Democratic Republic of the Congo [63]. Despite its value, however, this new protected area may provide an incomplete representation of priority timber species, as most of the historically known populations are located outside the Park boundaries. Eventual refinements to the limits of the Park may thus be desirable, which, together with prevention of illegal logging, could greatly assist in the protection of threatened timber tree species in Cabinda's Maiombe forest.

Urgent consideration should also be given to dry forests of Angola, where there are at least three timber species of high conservation priority, and where deforestation rates are increasing rapidly [27]. This is in line with the growing perception that tropical dry forests should be given high conservation priority, as they have a high biodiversity value in Sub-Saharan Africa [64], [65]. The current exploitation of dry forest trees of high conservation priority is unknown in Angola, but in the past they were all valued timber species [43], and they are exploited elsewhere in Africa [24]. Although some of these species may be represented in protected areas in the south of Angola, the degree of on-the-ground protection that these areas presently afford is very uncertain. It is thus recommended that particular conservation attention should be given to timber trees from dry forests, in the context of ongoing efforts to strengthen the network of protected areas in Angola [25], [33].

Lack of recent information is one of the key problems affecting biodiversity conservation in Angola. Shortage of data is probably more serious in Angola than in most places elsewhere in Africa, because of the prolonged war of independence (1961–1974) and post-independence civil war (1975–2002) which left the country largely inaccessible to most researchers. In these circumstances, studies such as the present one, based on accumulated historical information, may provide initial guidance on the identification of conservation priorities and problems, providing useful insights that would otherwise be very difficult to obtain [1], [2], [5], [6], [66]. However, it is now more than one decade after the end of the conflicts in Angola and, given the growing prosperity and development in the country, it is essential that new field surveys are undertaken to document contemporary species distributions and conservation challenges. In the particular case of tree species, it is also essential to collect quantitative information on species identity and places of origin of timber exports, which can then be used to guide new surveys and conservation assessments. Collecting this information would be essential to provide a solid basis for the conservation and sustainable use of forest resources in Angola.

## Supporting Information

Figure S1 **Temporal profile of herbarium records of the selected timber species of Angola.** For each species, the temporal range of herbarium specimens housed in the selected herbaria is indicated (grey horizontal line).
(TIF)

Figure S2 **Raster data of Maiombe forest cover in the lower Congo basin, showing Cabinda (Angola) and adjacent areas of Congo (upper) and Democratic Republic of the Congo (lower).** The pin bullet indicates a transition where a change in the forest cover density across the border is identified, with the higher density being on the Cabinda side. Maps were produced using data available on-line from: http://earthenginepartners.appspot.com/science-2013-global-forest ([27] Hansen et al. 2013. High-Resolution Global Maps of 21st-Century Forest Cover Change. Science 342. 850–853).
(TIF)

Table S1 **Georeferenced vouchers or bibliographic records for selected timber species in Angola.** Data from the LISC Herbarium is available through GBIF at http://www.gbif.org/dataset/231c5bcf-1b56-4905-a398-6d0e18f6de1a.
(DOC)

Table S2 **Sixty-two datasets from GBIF providers queried for the 18 timber species, producing 2253 records. Accessed 29 April 2013.**
(DOC)

Table S3 **Data provider per species accessed through GBIF data portal for each of the 18 timber species considered in this study. Accessed 29 April 2013.**
(DOC)

Table S4 **Characteristics of the 18 timber species studied, including information about their family, synonymy, common names, habit and ecology, and timber characteristics and uses.**
(DOC)
