# Supplementary figures and images for: Documenting Biogeographical Patterns of African Timber Species Using Herbarium Records: A Conservation Perspective Based on Native Trees from Angola
Source: PLoS One. 2014 Jul 25;9(7):e103403. doi: 10.1371/journal.pone.0103403 (PMC4111583; doi:10.1371/journal.pone.0103403)

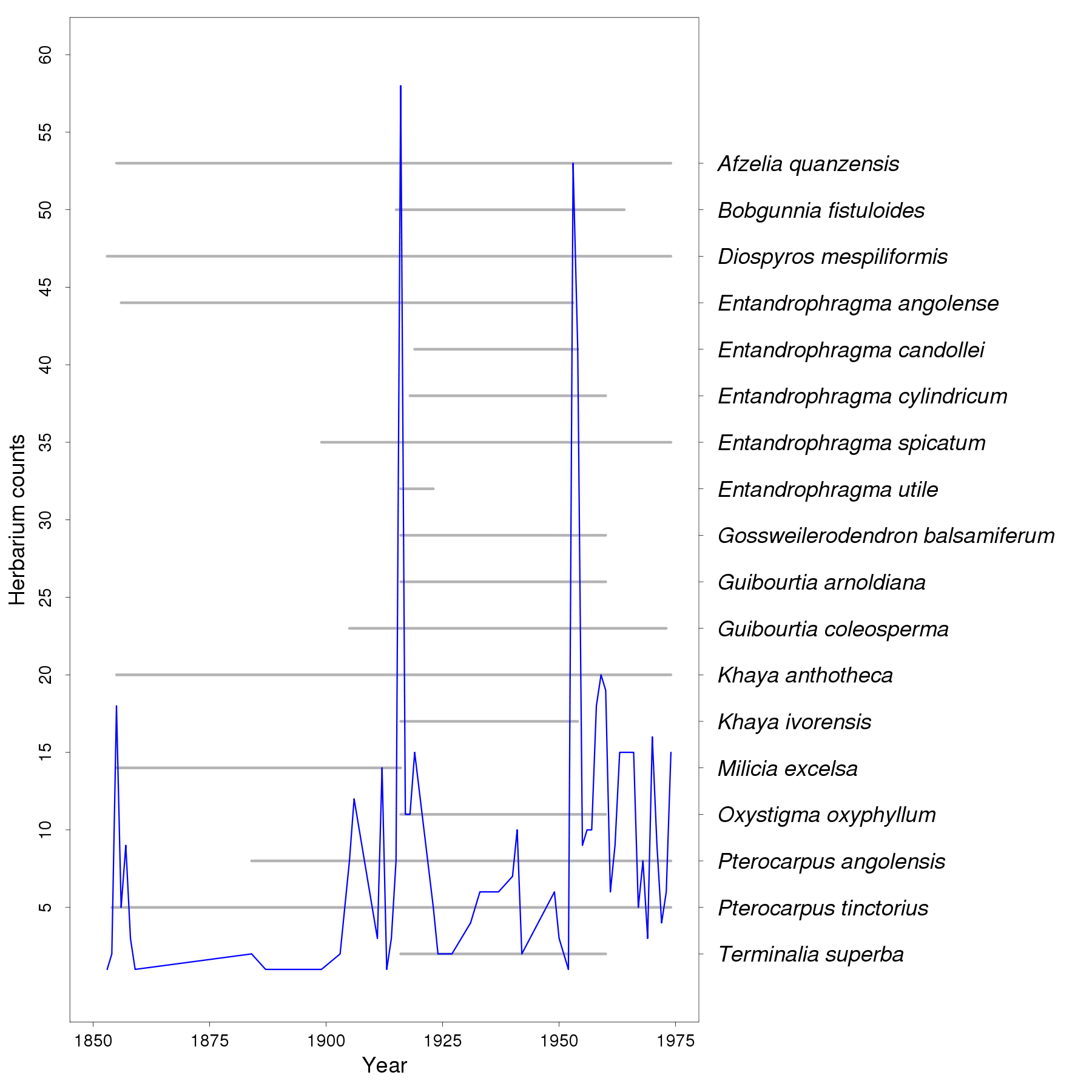

Supplement: Figure S1 — Temporal profile of herbarium records of the selected timber species of Angola. For each species, the temporal range of herbarium specimens housed in the selected herbaria is indicated (grey horizontal line). (TIF) [file pone.0103403.s001.tif]

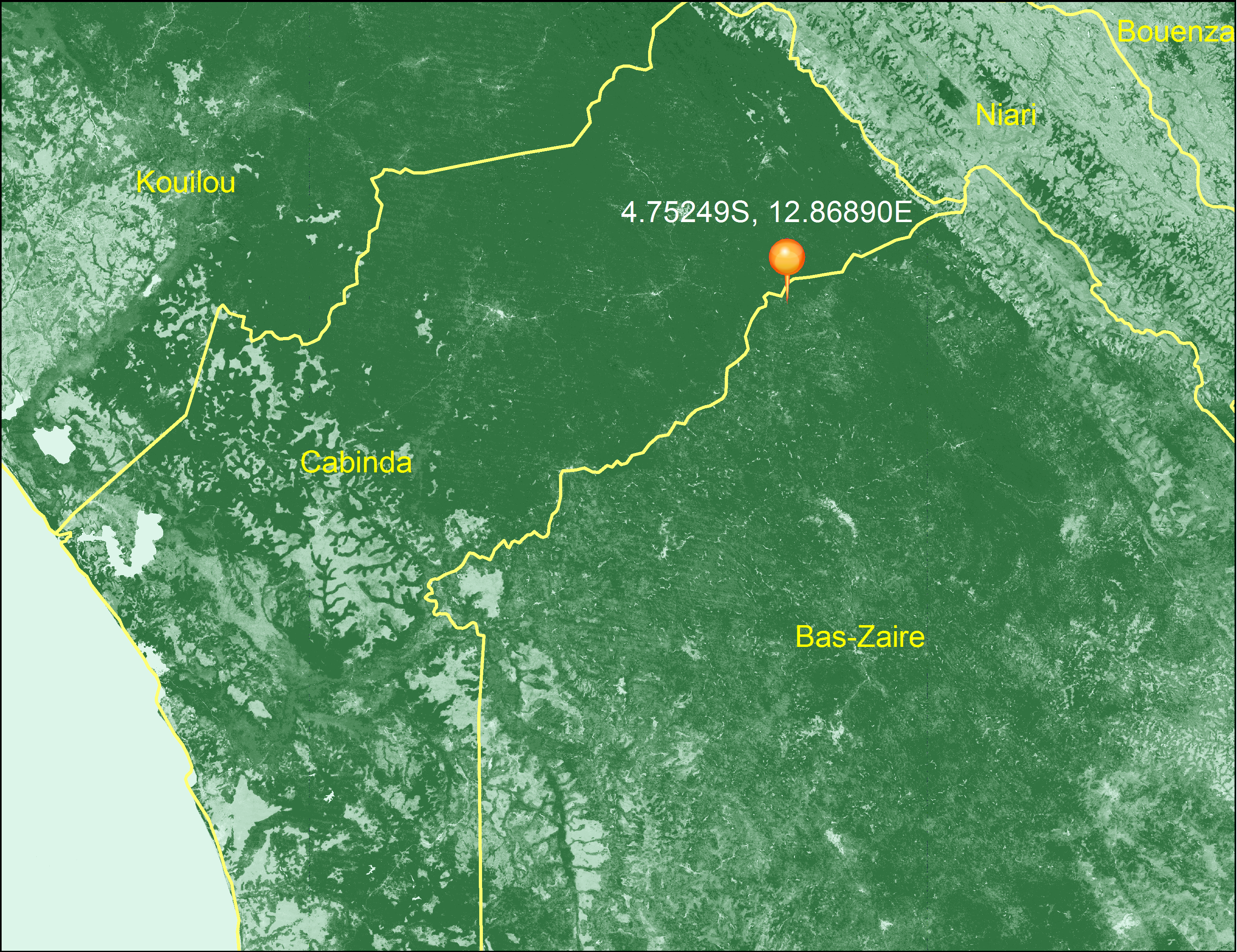

Supplement: Figure S2 — Raster data of Maiombe forest cover in the lower Congo basin, showing Cabinda (Angola) and adjacent areas of Congo (upper) and Democratic Republic of the Congo (lower). The pin bullet indicates a transition where a change in the forest cover density across the border is identified, with the higher density being on the Cabinda side. Maps were produced using data available on-line from: http://earthenginepartners.appspot.com/science-2013-global-forest ([27] Hansen et al. 2013. High-Resolution Global Maps of 21st-Century Forest Cover Change. Science 342. 850–853). (TIF) [file pone.0103403.s002.tif]
