# Supplementary material for: Documenting Biogeographical Patterns of African Timber Species Using Herbarium Records: A Conservation Perspective Based on Native Trees from Angola
Source: PLoS One. 2014 Jul 25;9(7):e103403. doi: 10.1371/journal.pone.0103403 (PMC4111583; doi:10.1371/journal.pone.0103403)
Supplement: Table S1 — Georeferenced vouchers or bibliographic records for selected timber species in Angola. Data from the LISC Herbarium is available through GBIF at http://www.gbif.org/dataset/231c5bcf-1b56-4905-a398-6d0e18f6de1a. (DOC) [file pone.0103403.s003.doc]

**Table S1.** Georeferenced vouchers or bibliographic records for selected timber species in Angola. Data from the LISC Herbarium is available through GBIF at http://www.gbif.org/dataset/231c5bcf-1b56-4905-a398-6d0e18f6de1a

| **Species** | **District** | **Locality** | **Colector** | **Herbarium** | **Longitude** | **Latitude** |
| --- | --- | --- | --- | --- | --- | --- |
| *Afzelia quanzensis* | BENGUELA | Ganda, Posto experimental do Sisal | Teixeira & Andrade 5030 | COI; LISC | 14.55 | -13.06667 |
| *Afzelia quanzensis* | BENGUELA | Cubal, entre Caimbambo e o Cubal, a 8km Caimbambo | Barbosa 11760 | LISC | 14 | -13.03333 |
| *Afzelia quanzensis* | BENGUELA | Estação Zootécnica (Ganda) | Araújo 816 | LISC | 14.5 | -12.93333 |
| *Afzelia quanzensis* | BENGUELA | Região do Cubal | Lains & Silva 8 | LISC | 14.31667 | -13.03333 |
| *Afzelia quanzensis* | BIÉ | Cuemba | R.Monteiro & Murta 1899 | COI; LISC | 18.1 | -12.13333 |
| *Afzelia quanzensis* | BIÉ | N´harea | R.Monteiro & Murta 2050 | COI; LISC | 17.05 | -11.41667 |
| *Afzelia quanzensis* | CUANDO-CUBANGO | Mavinga (Kiongozi) | R.Santos 2340 | LISC | 20.33333 | -15.75 |
| *Afzelia quanzensis* | CUANDO-CUBANGO | Mavinga (Luirongozi), entre o rio Luengue e o rio Cuito | R.Santos 2416 | LISC | 19.53333 | -16.68333 |
| *Afzelia quanzensis* | CUANDO-CUBANGO | N´Riquinha | Teixeira 51 | LISC | 21.7 | -15.96667 |
| *Afzelia quanzensis* | CUANDO-CUBANGO | Cuangar, Caiundo | R.Santos 2508 | LISC | 17.45 | -15.58333 |
| *Afzelia quanzensis* | CUANZA NORTE | N´Dalatando, Granja de S. Luís | Gossweiler 5944 | BM; COI; LISC | 14.9 | -9.31667 |
| *Afzelia quanzensis* | CUANZA NORTE | Golungo Alto, perto de Songue! | Welwitsch 593 | BM; LISU | 14.66667 | -9.11667 |
| *Afzelia quanzensis* | CUANZA NORTE | A 15km de Samba Cajou vers Carmona | Dechamps, Murta & M.Silva 1559 | LISC | 15.48333 | -8.73333 |
| *Afzelia quanzensis* | CUANZA NORTE | Cassoalala Luinha a 20km (na estrada) | Raimundo, Matos & Figueira 231 | LISC | 14.48333 | -9.23333 |
| *Afzelia quanzensis* | CUANZA SUL | Entre Chicunda e Lumete | Raimundo, Matos & Figueira 1138 | LISC | 15.01667 | -11.21667 |
| *Afzelia quanzensis* | CUNENE | Perto de Cuvelai entre Ondjiva e Cubango | Dechamps, Murta & M.Silva 1302 | LISC | 15.81667 | -15.63333 |
| *Afzelia quanzensis* | HUÍLA | Entre os ribeiros de Colui e Tanga, Ganguelas | Gossweiler 2120 | BM; COI; K; LISC | 16 | -14.5 |
| *Afzelia quanzensis* | HUÍLA | Humpata, escarpas das vertentes da Serra de Chela | Gossweiler 12783a | COI; LISC | 13.3 | -15 |
| *Afzelia quanzensis* | HUÍLA | A 15km de Quilengues para Impulo | Mendes 721 | LISC | 13.98333 | -14.01667 |
| *Afzelia quanzensis* | HUÍLA | Mulondo, na estrada para Capelongo ao km 31 | Menezes 804 | LISC | 15.26667 | -15.41667 |
| *Afzelia quanzensis* | HUÍLA | Gambos, Chibemba no Chimbolelo | Menezes 714 | LISC | 14.25 | -16 |
| *Afzelia quanzensis* | HUÍLA | Cassinga junto à residência do Chefe do Posto | Menezes1256 | LISC | 16.08333 | -15.13333 |
| *Afzelia quanzensis* | HUÍLA | Ganguelas, ao km 15 da picada Caiundo-Indungo | Menezes1198 | LISC | 16.51667 | -14.96667 |
| *Afzelia quanzensis* | HUÍLA | Lubango | Teixeira & Andrade 432 | LISC | 13.5 | -14.91667 |
| *Afzelia quanzensis* | MALANGE | Hemisilva na área de Malange | C.T.Almeida s.n. | LISC | 16.5 | -9.5 |
| *Afzelia quanzensis* | MALANGE | Malange | Gossweiler s.n. | BM, col. carp. | 16.33333 | -9.53333 |
| *Afzelia quanzensis* | MALANJE | Pungo Andongo, margens do rio Cuanza | Welwitsch 594 | BM; COI; LISU | 15.63333 | -9.7 |
| *Afzelia quanzensis* | MALANJE | Malanje, Banza de Bumba, prox. de Sange | Welwitsch 628 | BM; COI; LISU | 15.56667 | -9.28333 |
| *Afzelia quanzensis* | NAMIBE | Bibala, escarpas do sopé da Chela | Gossweiler 12783 | LISC | 13.25 | -14.83333 |
| *Afzelia quanzensis* | NAMIBE | Serra de Chela, Bibala | Torre 12783 | LISC | 13.16667 | -14.83333 |
| *Afzelia quanzensis* | NAMIBE | Entre Camucuio e Quilengues, Lola | Mendonça 4540 | LISC | 13.58333 | -14.28333 |
| *Afzelia quanzensis* | NAMIBE | Entre Camucuio e Quilengues, Lola | Mendonça 4542 | LISC | 13.58333 | -14.21667 |
| *Afzelia quanzensis* | NAMIBE | Bibala, Lola | Teixeira & Andrade 4270 | COI; LISC | 13.58333 | -14.28333 |
| *Afzelia quanzensis* | NAMIBE | Lungo, Maconge | Teixeira 686 | COI; LISC | 13.2 | -14.31667 |
| *Afzelia quanzensis* | NAMIBE | Tampa, Fazenda Nova Fé | Correia 1847 | LISC | 13.18333 | -15.01667 |
| *Bobgunnia fistuloides* | CABINDA | Maiombe, nas margens do rio Luali | Gossweiler 6265b | LISC | 12.48333 | -5.08333 |
| *Bobgunnia fistuloides* | CABINDA | Buco Zau, Chiaca | MEFA-Carvalho 392 | LISC | 12.53333 | -4.95 |
| *Bobgunnia fistuloides* | CABINDA | Buco Zau, prox. do Morro do Cuidado | R.Monteiro, R.Santos & Murta 360 | COI; LISC | 12.55 | -4.76667 |
| *Bobgunnia fistuloides* | CABINDA | Buco Zau, Chiaca | Teixeira & Gerez 7514 | LISC | 12.53333 | -4.95 |
| *Bobgunnia fistuloides* | CABINDA | Buco Zau, Chiaca | Frade 10 | LISC | 12.53333 | -4.95 |
| *Bobgunnia fistuloides* | CUANZA NORTE | Ilha Calemba no rio Cuanza, prox. de Quissonde | Gossweiler s.n | LISC | 16.31667 | -9.96667 |
| *Bobgunnia fistuloides* | ZAIRE | S. António do Zaire | Gossweiler s.n | LISC | 12.33333 | -6.15 |
| *Diospyros mespiliformis* | BENGO | Entre a Muxima e a fazenda Mucumbi | Raimundo, Matos & Figueira 571 | LISC | 13.95 | -9.5 |
| *Diospyros mespiliformis* | BENGO | Entre Mumbondo e Quixinge Velho, andados 37km | Raimundo, Matos & Figueira 595 | LISC | 14.3 | -9.93333 |
| *Diospyros mespiliformis* | BENGO | Baixa dos elefantes, Parque Nacional da Quiçaqma | Teixeira & al. 11989 | LISC | 13.5 | -9.75 |
| *Diospyros mespiliformis* | BENGO | Ambriz, entre Ambriz e Mossul | Welwitsch 2528 | LISU | 13.28333 | -7.9 |
| *Diospyros mespiliformis* | BENGO | Catete | Gossweiler 9190 | LISC | 13.68333 | -9.1 |
| *Diospyros mespiliformis* | CUANDO-CUBANGO | Ao km 52 da picada Caiundo-Indungo | Menezes1206 | LISC | 17.11667 | -15.33333 |
| *Diospyros mespiliformis* | CUANDO-CUBANGO | Posto do Luiana | Teixeira 85 | LISC | 23 | -17.35 |
| *Diospyros mespiliformis* | CUANDO-CUBANGO | Cuangar. Caiundo | R.Santos 2507 | LISC | 17.45 | -15.58333 |
| *Diospyros mespiliformis* | CUANDO-CUBANGO | Proximo da Foz do Cuatir, entre Caíra e Savate | Matos 130 | LISC | 18.13333 | -16.83333 |
| *Diospyros mespiliformis* | CUANZA NORTE | Na junção do rio Mucoso e a estrada N´Dalatando-Dondo | Dechamps, Murta & M.Silva 1571 | LISC | 14.7 | -9.58333 |
| *Diospyros mespiliformis* | CUANZA SUL | Bumba, na margem do rio Gango | Welwitsch 2529 | LISU | 15.7 | -9.9 |
| *Diospyros mespiliformis* | CUANZA SUL | Zona de N´Gunza. A caminho de Carimba | Teixeira & al. 11538 | LISC | 13.86667 | -11.06667 |
| *Diospyros mespiliformis* | CUANZA SUL | Cachoeiras-Bimbe ao km20 | Teixeira & al. 11303 | LISC | 14.75 | -11.03333 |
| *Diospyros mespiliformis* | CUNENE | Curoca, Chitado, prox. do Ruacaná | Menezes & Henriques 107 | LISC | 14.2 | -17.31667 |
| *Diospyros mespiliformis* | CUNENE | Curoca, Chitado | Menezes & Henriques 54 | LISC; LISU | 13.91667 | -17.25 |
| *Diospyros mespiliformis* | CUNENE | Cahama, Mupa, arredores | Menezes 1406 | LISC | 15.73333 | -16.16667 |
| *Diospyros mespiliformis* | CUNENE | Cuamato, arredores do Humbe | Sousa 34 | LISC | 14.9 | -16.61667 |
| *Diospyros mespiliformis* | CUNENE | Mucope, Humbe | Henriques 212 | LISC | 14.91667 | -16.18333 |
| *Diospyros mespiliformis* | CUNENE | Ondjiva, prox. de Chiede | Mendonça 4551 | LISC | 15.91667 | -17 |
| *Diospyros mespiliformis* | CUNENE | Ondjiva | Mendonça 4561 | LISC | 15.71667 | -17 |
| *Diospyros mespiliformis* | CUNENE | Xangongo. Centro Estudos Cunene. Margens rio Cunene | Raimundo & al. s.n | LISC | 15.03333 | -16.65 |
| *Diospyros mespiliformis* | CUNENE | Ondjiva- Cubango km 26 | Bamps, Raimundo & Matos 4088 | LISC | 15.83333 | -16.91667 |
| *Diospyros mespiliformis* | CUNENE | Perto do rio Cunene | Dechamps, Murta & M.Silva 1271 | LISC | 15.11667 | -16.7 |
| *Diospyros mespiliformis* | CUNENE | Xangongo, na margem direita do rio Cunene | Menezes 3456 | LISC | 15.01667 | -16.65 |
| *Diospyros mespiliformis* | CUNENE | Xangongo, na margem do rio Cunene, prox. de Humbe | R.Santos & Barroso 2704 | LISC | 14.98333 | -16.63333 |
| *Diospyros mespiliformis* | CUNENE | Ondjiva, arredores | Barroso & Correia 9025 | LISC | 15.71667 | -17 |
| *Diospyros mespiliformis* | HUÍLA | Mulondo, na picada para Quiteve ao km24 | Menezes 775 | LISC | 15.2 | -15.96667 |
| *Diospyros mespiliformis* | HUÍLA | Quihita | Antunes & Dekindt 460 | LISC | 13.96667 | -15.36667 |
| *Diospyros mespiliformis* | HUÍLA | Mulondo, margens do Cunene | Barbosa 10763 | LISC | 15.18333 | -15.58333 |
| *Diospyros mespiliformis* | HUÍLA | Huíla | Exell & Mendonça 2707 | LISC | 13.53333 | -15.06667 |
| *Diospyros mespiliformis* | HUÍLA | Huíla | Exell & Mendonça 2325 | LISC | 13.53333 | -15.06667 |
| *Diospyros mespiliformis* | LUANDA | A 2km da foz do rio Cuanza | R.Santos 1439 | LISC | 13.18333 | -9.31667 |
| *Diospyros mespiliformis* | LUANDA | Viana. Vale do Bengo. | Teixeira & al. 10164 | LISC | 13.46667 | -8.86667 |
| *Diospyros mespiliformis* | NAMIBE | Base da Serra de Chela, de Bruco para Mumpula | Welwitsch 2530 | LISU | 13.36667 | -15.13333 |
| *Diospyros mespiliformis* | NAMIBE | Serra de Chela, Bibala | Gossweiler 13222 | LISC | 13.16667 | -14.83333 |
| *Diospyros mespiliformis* | NAMIBE | Serra de Chela, Bibala | Gossweiler 12957 | LISC | 13.16667 | -14.83333 |
| *Diospyros mespiliformis* | NAMIBE | Na margem do rio Santa Tereza, entre Munhino e Bruco | Mendes 956 | LISC | 13.11667 | -14.88333 |
| *Diospyros mespiliformis* | NAMIBE | Cainde, margem do rio Cubal | R.Santos 1 | LISC | 13.43333 | -15.68333 |
| *Diospyros mespiliformis* | NAMIBE | Camucuio, ao km5 da estrada Camucuio-Lungo | Menezes 3140 | LISC | 13.23333 | -14.15 |
| *Diospyros mespiliformis* | NAMIBE | Bibala, Lungo | Henriques & Moreno 24 | LISC | 13.21667 | -14.31667 |
| *Entandrophragma angolense* | CABINDA | Bôma-Lubinda | Moura 465 | LISC | 12.13333 | -5.03333 |
| *Entandrophragma angolense* | CABINDA | Maiombe, Buco Zau | Gossweiler 8088 | BM; COI; K; LISC; LISU | 12.55 | -4.76667 |
| *Entandrophragma angolense* | CABINDA | Maiombe, Buco Zau | Gossweiler 6800 | BM; COI; K; LISC; LISU | 12.55 | -4.76667 |
| *Entandrophragma angolense* | CABINDA | Buco Zau, rio Lubambe (Zubambe) | Gossweiler 6501 | BM; COI; LISC; LISU | 12.55 | -4.76667 |
| *Entandrophragma angolense* | CABINDA | Prox. da confluência do Luali com o Chiluango | Gossweiler 9086 | B; BM | 12.48333 | -5.05 |
| *Entandrophragma angolense* | CABINDA | Prox. da confluência do Luali com o Chiluango | Gossweiler 9086b | BM, col.carp.; K; LISC | 12.48333 | -5.05 |
| *Entandrophragma angolense* | CUANZA NORTE | Golungo Alto. Serra do Alto Queta | Welwitsch 1313 | BM; COI; K; LISC; LISU | 14.76667 | -9.26667 |
| *Entandrophragma angolense* | CUANZA NORTE | Cazengo, Granja de S. Luís | Gossweiler 5849 | LISU | 14.9 | -9.31667 |
| *Entandrophragma angolense* | CUANZA NORTE | Cazengo, N´Dalatando | Gossweiler 10136 | COI | 14.91667 | -9.3 |
| *Entandrophragma angolense* | CUANZA SUL | Amboim, Capir | Gossweiler 10057 | B; K; LISC | 14.3 | -10.75 |
| *Entandrophragma angolense* | MALANGE | Bondo e Bângala, Quela | Nolde 208 | B | 17.11667 | -9.2 |
| *Entandrophragma candollei* | CABINDA | Maiombe, Buco Zau | Gossweiler 7237 | BM; COI; LISC; LISU | 12.55 | -4.76667 |
| *Entandrophragma candollei* | CABINDA | Maiombe, Buco Zau | Gossweiler 7320 | BM; COI; LISU | 12.55 | -4.76667 |
| *Entandrophragma candollei* | CABINDA | Maiombe, Buco Zau | Gossweiler 7535 | BM; COI; LISU | 12.55 | -4.76667 |
| *Entandrophragma candollei* | CABINDA | Maiombe, Buco Zau, Monte do mogno | Gossweiler 7537 | BM; LISC; LISU | 12.55 | -4.76667 |
| *Entandrophragma candollei* | CABINDA | Maiombe, Buco Zau | Gossweiler 8078 | B; COI; LISC; LISU | 12.55 | -4.76667 |
| *Entandrophragma candollei* | CABINDA | Maiombe, Buco Zau | Gossweiler s.n. | BM | 12.55 | -4.76667 |
| *Entandrophragma cylindricum* | CABINDA | Maiombe, Buco Zau | Gossweiler 6684 | BM; LISU | 12.55 | -4.76667 |
| *Entandrophragma cylindricum* | CABINDA | Maiombe, Buco Zau | Gossweiler 7535 | BM; COI; LISC; LISU | 12.55 | -4.76667 |
| *Entandrophragma cylindricum* | CABINDA | Maiombe, Buco Zau | Gossweiler 7538 | COI | 12.55 | -4.76667 |
| *Entandrophragma cylindricum* | CABINDA | Maiombe, Buco Zau | Gossweiler 7548 | B; BM; COI; LISC; LISU | 12.55 | -4.76667 |
| *Entandrophragma cylindricum* | CABINDA | Maiombe, Buco Zau | Gossweiler 8131 | BM; COI; LISC; LISU | 12.55 | -4.76667 |
| *Entandrophragma cylindricum* | CABINDA | Maiombe, Buco Zau | Gossweiler 8087 | BM; COI; LISU | 12.55 | -4.76667 |
| *Entandrophragma cylindricum* | CABINDA | Ncanda Mbaco | Gossweiler 9083 | BM, col. carp. | 12.48333 | -5.05 |
| *Entandrophragma cylindricum* | CABINDA | Maiombe, Buco Zau | MEFA- D´Orey 660 | LISC | 12.55 | -4.76667 |
| *Entandrophragma cylindricum* | CABINDA | Pango Mongo, Sub-Luali | Gossweiler 8031 | BM; COI; LISC; LISU | 12.46667 | -5.08333 |
| *Entandrophragma spicatum* | BENGUELA | Estrada Ganda Cubal, a cerca de 2km da última | Raimundo, Matos & Figueira 1334 | LISC | 14.26667 | -12.96667 |
| *Entandrophragma spicatum* | BENGUELA | Entre Cubal e Caimbambo | Dechamps, Murta & M.Silva 1087 | LISC | 14.16667 | -13.05 |
| *Entandrophragma spicatum* | BENGUELA | Perto de Caimbambo | Dechamps, Murta & M.Silva 1096 | LISC | 14 | -13.03333 |
| *Entandrophragma spicatum* | BENGUELA | Perto de Caimbambo | Dechamps, Murta & M.Silva 1099 | LISC | 14 | -13.03333 |
| *Entandrophragma spicatum* | BENGUELA | Estrada Ganda-Cubal ao km13 | Bamps, S.Martins & Matos 4447 | LISC | 14.33333 | -13 |
| *Entandrophragma spicatum* | CUNENE | Ondjiva | Baum 88 | BM; COI; K | 15.71667 | -17 |
| *Entandrophragma spicatum* | CUNENE | Xangongo, entre as picadas Dimba-Catequero-Humbe | R.Santos, Schultz & Filipe 2536 | LISC | 15 | -16.68333 |
| *Entandrophragma spicatum* | CUNENE | Xangongo | Murta & M. Silva 775 | LISC | 15 | -16.75 |
| *Entandrophragma spicatum* | CUNENE | Cahama, Mupa, no morro Lucambo | Menezes 1346 | LISC | 15.73333 | -16.16667 |
| *Entandrophragma spicatum* | CUNENE | Ondjiva, prox. de Chiede | Mendonça 4558 | LISC | 15.91667 | -17 |
| *Entandrophragma spicatum* | CUNENE | Xangongo, Humbe, entre Humbe e Catequero | R.Santos & Barroso 2824 | LISC | 14.93333 | -16.61667 |
| *Entandrophragma spicatum* | CUNENE | Xangongo, Humbe, entre Humbe e Catequero | R.Santos & Barroso 2835 | LISC | 14.93333 | -16.61667 |
| *Entandrophragma spicatum* | CUNENE | A 35km de Ondjiva para Xangongo | Dechamps, Murta & M.Silva 1278 | LISC | 15.5 | -16.95 |
| *Entandrophragma spicatum* | CUNENE | Entre Xangongo e Môngua andados 5km | Raimundo, Matos & Figueira 1175 | LISC | 15.08333 | -16.75 |
| *Entandrophragma spicatum* | CUNENE | Ao km15 de Cahama, estrada de Gambos | Torre 8749 | LISC | 14.21667 | -16.18333 |
| *Entandrophragma spicatum* | CUNENE | Posto Zootecnico do Cafú (cunene) | Teixeira 2592 | LISC | 15.28333 | -16.3 |
| *Entandrophragma spicatum* | HUÍLA | Quilengues | Teixeira 630 | LISC | 14.06667 | -14.08333 |
| *Entandrophragma spicatum* | HUÍLA | Alto cunene, Mulondo (arredores) | Menezes 821 | LISC | 15.23333 | -15.65 |
| *Entandrophragma spicatum* | NAMIBE | Serra de Chela, Bibala | Gossweiler13375 | LISC | 13.16667 | -14.83333 |
| *Entandrophragma spicatum* | NAMIBE | Bibala | Gossweiler 13266 | LISC | 13.26667 | -14.76667 |
| *Entandrophragma spicatum* | NAMIBE | Bibala, escarpas do sopé da Chela | Dekindt 1169 | LISC | 13.25 | -14.83333 |
| *Entandrophragma spicatum* | NAMIBE | Bibala , Lungo | Teixeira 664 | LISC | 13.21667 | -14.31667 |
| *Entandrophragma spicatum* | NAMIBE | Estrada da Serra de Chela prox. de Humbia | Teixeira 2414 | LISC | 13.45 | -14.73333 |
| *Entandrophragma utile* | CABINDA | Maiombe, Buco Zau | Gossweiler 6695 | BM; LISC; LISU | 12.55 | -4.76667 |
| *Entandrophragma utile* | CABINDA | Maiombe, Buco Zau | Gossweiler 6729 | COI; LISC; LISU | 12.55 | -4.76667 |
| *Entandrophragma utile* | CABINDA | Maiombe, Buco Zau | Gossweiler 6758 | BM | 12.55 | -4.76667 |
| *Entandrophragma utile* | CABINDA | Maiombe, Buco Zau | Gossweiler 8087 | LISC | 12.55 | -4.76667 |
| *Entandrophragma utile* | CABINDA | Maiombe, Buco Zau | Gossweiler 6790 | BM; LISC; LISU | 12.55 | -4.76667 |
| *Entandrophragma utile* | CABINDA | Maiombe, Buco Zau | Gossweiler 7332 | BM; COI; LISC; LISU | 12.55 | -4.76667 |
| *Entandrophragma utile* | CABINDA | Maiombe, Buco Zau | Gossweiler 7538 | BM; COI; LISC; LISU | 12.55 | -4.76667 |
| *Entandrophragma utile* | CABINDA | Rio Lubambe, Buco Zau | Gossweiler 8073 | BM; COI; K; LISC | 12.55 | -4.76667 |
| *Entandrophragma utile* | CABINDA | Ncanda Mbaco | Gossweiler 9085 | B; BM; K; LISC | 12.48333 | -5.05 |
| *Entandrophragma utile* | CABINDA | Ncanda Mbaco | Gossweiler 9085b | BM col. carp; B | 12.48333 | -5.05 |
| *Gossweilerodendron balsamiferum* | CABINDA | Maiombe | Dawe 240 | K | 12.58333 | -4.73333 |
| *Gossweilerodendron balsamiferum* | CABINDA | Maiombe, Buco Zau | Gossweiler 7249 | BM; COI; K; LISC; LISU | 12.55 | -4.76667 |
| *Gossweilerodendron balsamiferum* | CABINDA | Morro do Mungo Vitala, perto de Buco Zau | Gossweiler 7249b | LISC | 12.5 | -4.66667 |
| *Gossweilerodendron balsamiferum* | CABINDA | Maiombe, Buco Zau | Gossweiler 7258 | BM; COI; K; LISC; LISU | 12.55 | -4.76667 |
| *Gossweilerodendron balsamiferum* | CABINDA | Ncanda Mbaco, prox. do Luali com o Chiluango | Gossweiler 7258b | K; LISC | 12.48333 | -5.05 |
| *Gossweilerodendron balsamiferum* | CABINDA | Maiombe, Buco Zau | Gossweiler 7258c | LISC | 12.55 | -4.76667 |
| *Gossweilerodendron balsamiferum* | CABINDA | Ncanda Mbaco | Gossweiler 7258d | LISC | 12.48333 | -5.05 |
| *Gossweilerodendron balsamiferum* | CABINDA | Pango Mongo | Gossweiler 6192 | BM; COI; LISU | 12.5 | -5.03333 |
| *Gossweilerodendron balsamiferum* | CABINDA | Maiombe, Chiaca | Cameira 125 | LISC | 12.53333 | -4.95 |
| *Gossweilerodendron balsamiferum* | CABINDA | Maiombe, Chiaca (estrada para Buco Zau) | Murta 40 | COI; LISC | 12.53333 | -4.95 |
| *Gossweilerodendron balsamiferum* | CABINDA | Maiombe, Chiaca, Buco Zau | Frade 1 | LISC | 12.53333 | -4.95 |
| *Gossweilerodendron balsamiferum* | CABINDA | Bôma-Lubinda | Moura 477 | COI; LISC | 12.13333 | -5.03333 |
| *Gossweilerodendron balsamiferum* | CABINDA | Bôma-Lubinda | A. Silva 892 | COI; LISC | 12.13333 | -5.03333 |
| *Gossweilerodendron balsamiferum* | CABINDA | Bôma-Lubinda | A. Silva 888 | COI; LISC | 12.13333 | -5.03333 |
| *Gossweilerodendron balsamiferum* | CABINDA | Bôma-Lubinda | A. Silva 889 | COI; LISC | 12.13333 | -5.03333 |
| *Gossweilerodendron balsamiferum* | CABINDA | Bôma-Lubinda (Dinge) | A. Silva 495 | COI; LISC | 12.36667 | -4.96667 |
| *Gossweilerodendron balsamiferum* | CABINDA | Bôma-Lubinda (Dinge) | A. Silva 496 | COI; LISC | 12.36667 | -4.96667 |
| *Gossweilerodendron balsamiferum* | CABINDA | Bôma-Lubinda (Dinge) | A. Silva 501 | COI; LISC | 12.36667 | -4.96667 |
| *Gossweilerodendron balsamiferum* | CABINDA | Bôma-Lubinda (Dinge) | A. Silva 491 | COI; LISC | 12.36667 | -4.96667 |
| *Gossweilerodendron balsamiferum* | CABINDA | Bôma-Lubinda (Dinge) | A. Silva 490 | COI; LISC | 12.36667 | -4.96667 |
| *Gossweilerodendron balsamiferum* | CABINDA | Bôma-Lubinda (Dinge) | A. Silva 886 | LISC | 12.36667 | -4.96667 |
| *Gossweilerodendron balsamiferum* | CABINDA | Bôma-Lubinda (Dinge) | A. Silva 479 | COI; LISC | 12.36667 | -4.96667 |
| *Gossweilerodendron balsamiferum* | CABINDA | Bôma-Lubinda (Dinge) | A. Silva 483 | COI; LISC | 12.36667 | -4.96667 |
| *Gossweilerodendron balsamiferum* | CABINDA | Bôma-Lubinda (Dinge) | A. Silva 484 | COI; LISC | 12.36667 | -4.96667 |
| *Gossweilerodendron balsamiferum* | CABINDA | Bôma-Lubinda (Cacongo) | A. Silva 887 | COI; LISC | 12.13333 | -5.21667 |
| *Gossweilerodendron balsamiferum* | CABINDA | Bôma-Lubinda (Cacongo) | A. Silva 877 | LISC | 12.13333 | -5.21667 |
| *Gossweilerodendron balsamiferum* | CABINDA | Bôma-Lubinda (Cacongo) | A. Silva 890 | COI; LISC | 12.13333 | -5.21667 |
| *Gossweilerodendron balsamiferum* | CABINDA | Bôma-Lubinda (Cacongo) | A. Silva 893 | COI; LISC | 12.13333 | -5.21667 |
| *Gossweilerodendron balsamiferum* | CABINDA | Bôma-Lubinda (Cacongo) | A. Silva 891 | COI; LISC | 12.13333 | -5.21667 |
| *Gossweilerodendron balsamiferum* | CABINDA | Maiombe | MEFA -Carvalho 118 | LISC | 12.58333 | -4.73333 |
| *Gossweilerodendron balsamiferum* | CABINDA | Maiombe | MEFA -Carvalho 107 | LISC | 12.58333 | -4.73333 |
| *Gossweilerodendron balsamiferum* | CABINDA | Maiombe | MEFA -Carvalho 110 | LISC | 12.58333 | -4.73333 |
| *Gossweilerodendron balsamiferum* | CABINDA | Maiombe | MEFA -D´Orey 811 | LISC | 12.58333 | -4.73333 |
| *Guibourtia arnoldiana* | CABINDA | Maiombe, Chiluango | Gossweiler 6245 | K | 12.11667 | -5.2 |
| *Guibourtia arnoldiana* | CABINDA | Pango Mongo, Sub-Luali | Gossweiler 6470 | BM; COI; K; LISU; LISC | 12.46667 | -5.08333 |
| *Guibourtia arnoldiana* | CABINDA | Pango Mongo, Sub-Luali | Gossweiler 6470b | LISC | 12.46667 | -5.08333 |
| *Guibourtia arnoldiana* | CABINDA | Ncanda Mbaco, prox. da confluência do Luali-Chiluango | Gossweiler 8008 | K | 12.55 | -5.05 |
| *Guibourtia arnoldiana* | CABINDA | Ncanda Mbaco | Gossweiler 9008 | LISC | 12.55 | -5.05 |
| *Guibourtia arnoldiana* | CABINDA | Ncanda Mbaco, prox. da confluência do Luali-Chiluango | Gossweiler 9008b | BM; K | 12.55 | -5.05 |
| *Guibourtia arnoldiana* | CABINDA | Buco Zau, Reserva da Chiaca | R.Monteiro, R.Santos & Murta 323 | LISC | 12.53333 | -4.95 |
| *Guibourtia arnoldiana* | CABINDA | Buco Zau, Reserva da Chiaca | MEFA- D´Orey 671 | LISC | 12.53333 | -4.95 |
| *Guibourtia arnoldiana* | CABINDA | Buco Zau, Reserva da Chiaca | MEFA-Carvalho 330 | LISC | 12.53333 | -4.95 |
| *Guibourtia arnoldiana* | CABINDA | Buco Zau, Reserva da Chiaca | MEFA- D´Orey 608 | LISC | 12.53333 | -4.95 |
| *Guibourtia arnoldiana* | ZAIRE | Sumba, Peco | Gossweiler 9145 | BM, LISU | 12.75 | -6.13333 |
| *Guibourtia coleosperma* | BENGO | Icolo e Bengo | Pereira 296 | COI; LISC | 13.75 | -9 |
| *Guibourtia coleosperma* | BIÉ | Cuemba, prox. do rio Cuíva | R.Santos 1735 | LISC | 18.05 | -12.2 |
| *Guibourtia coleosperma* | CUANDO-CUBANGO | Entre os rios Cubango e Cuito | Baum 507 | BM; COI; K | 19.5 | -17.5 |
| *Guibourtia coleosperma* | CUANDO-CUBANGO | Entre Chipomba e Menongue | Gossweiler 2449 | BM; COI; K; LISC | 17.8 | -14.6 |
| *Guibourtia coleosperma* | CUANDO-CUBANGO | Entre os rios Luassinga e Longa | Gossweiler 2707 | BM; COI; K; LISC | 18.66667 | -15.66667 |
| *Guibourtia coleosperma* | CUANDO-CUBANGO | Rio Luassinga, Menongue | Gossweiler 2855 | BM; COI; K; LISC | 18.28333 | -14.75 |
| *Guibourtia coleosperma* | CUANDO-CUBANGO | Menongue, entre os rios Longa Luassinga | Gossweiler 3575 | BM; COI; K; LISC | 18.5 | -15.16667 |
| *Guibourtia coleosperma* | CUANDO-CUBANGO | Xamavera | Ponte & M. Silva 4110 | LISC | 20.36667 | -17.75 |
| *Guibourtia coleosperma* | CUANDO-CUBANGO | Menongue, perto da missão | Mendes 2258 | COI; LISC | 17.66667 | -14.63333 |
| *Guibourtia coleosperma* | CUANDO-CUBANGO | Cuito-Cuanavale, na Sede | Mendes 2952 | LISC | 19.18333 | -15.16667 |
| *Guibourtia coleosperma* | CUANDO-CUBANGO | Entre Caíra e Savate (Cuangar) | Matos 125 | LISC | 18.3 | -17.06667 |
| *Guibourtia coleosperma* | CUANDO-CUBANGO | Cuito-Cuanavale | Teixeira 28 | LISC | 19.21667 | -15.15 |
| *Guibourtia coleosperma* | CUANDO-CUBANGO | Na estrada de Menongue Catota | P.Almeida 381 | LISC | 17.68333 | -14.28333 |
| *Guibourtia coleosperma* | CUNENE | Cahama | Menezes 4016 | LISC | 14.31667 | -16.28333 |
| *Guibourtia coleosperma* | CUNENE | Ondjiva, na estrada Melunga ao km84 | Menezes 978 | LISC | 15.85 | -17 |
| *Guibourtia coleosperma* | CUNENE | Cahama, Mupa, arredores | Menezes 1330 | LISC | 15.73333 | -16.16667 |
| *Guibourtia coleosperma* | CUNENE | Cahama, Chiede, ao km 14 da picada para Mulemba | Menezes 1448 | LISC | 16.15 | -17.08333 |
| *Guibourtia coleosperma* | CUNENE | Chiede | Teixeira 3128 | LISC; COI | 16 | -17.16667 |
| *Guibourtia coleosperma* | HUAMBO | Huambo, Chitende | Andrada 26 | LISC; LISU; COI | 15.91667 | -11.71667 |
| *Guibourtia coleosperma* | HUILA | Caconda | Gossweiler s. n. | BR | 15.1 | -13.71667 |
| *Guibourtia coleosperma* | HUÍLA | Parque. Nac. do Bicuar | Teixeira & al 12595 | LISC | 14.75 | -15.25 |
| *Guibourtia coleosperma* | HUÍLA | Chibemba, na picada Chimbolele-Vimpôna km 39 | Menezes 617 | LISC | 15 | -15.41667 |
| *Guibourtia coleosperma* | HUÍLA | Alto cunene, Mulondo na picada para Bicuar km 28 | Menezes 889 | LISC | 15.08333 | -15.53333 |
| *Guibourtia coleosperma* | HUÍLA | Alto cunene, Mulondo para Chimbolelo km30 | Menezes 840 | LISC | 14.96667 | -15.68333 |
| *Guibourtia coleosperma* | LUNDA | Saurimo | Exell & Mendonça 580 | BM; COI; LISC | 20.4 | -9.65 |
| *Guibourtia coleosperma* | LUNDA | Saurimo | Gossweiler 11667 | COI; K | 20.4 | -9.65 |
| *Guibourtia coleosperma* | LUNDA | Saurimo | Lynes 305 | BR | 20.4 | -9.65 |
| *Guibourtia coleosperma* | LUNDA | Saurimo, sem loc. precisa | Lynes 274 | BR | 20.25 | -9.5 |
| *Guibourtia coleosperma* | LUNDA | Saurimo | V. Martins s.n. | LISC | 20.4 | -9.65 |
| *Guibourtia coleosperma* | LUNDA | A cerca de 45 km de Saurimo, na estrada Lucapa-Andrada | R.Santos 1654 | COI; LISC | 20.26667 | -9.46667 |
| *Guibourtia coleosperma* | LUNDA | Lucapa | Nº38-Veg.36 | LISC | 20.65 | -8.16667 |
| *Guibourtia coleosperma* | MOXICO | Cazombo | Samariamba 372-D | LISC | 22.86667 | -11.9 |
| *Guibourtia coleosperma* | MOXICO | Luena, a 4km de Luena ao longo do C:F | R.Monteiro, R.Santos & Murta 500 | LISC | 19.75 | -11.88333 |
| *Guibourtia coleosperma* | MOXICO | Luena, centro de estudos | Teixeira & Pedro 7545 | LISC; COI | 19.9 | -11.76667 |
| *Guibourtia coleosperma* | MOXICO | Luau | Gossweiler 12495 | LISC | 22.2 | -10.7 |
| *Guibourtia coleosperma* | MOXICO | Luau | Gossweiler 12496 | BM; LISC | 22.2 | -10.7 |
| *Khaya anthoteca* | BENGO | Luanda- Caxito, Ucua | R.Monteiro, R.Santos & Murta 187 | LISC | 14.16667 | -8.65 |
| *Khaya anthoteca* | BENGO | Dimuca. Entre Negage e Bengo andados 21km | Raimundo & al. 370 | LISC | 15.58333 | -8.11667 |
| *Khaya anthoteca* | CUANZA NORTE | Cazengo, N´Dalatando | Gossweiler 5974 | LISU; BM | 14.91667 | -9.3 |
| *Khaya anthoteca* | CUANZA NORTE | Cazengo, Granja de S. Luís | Gossweiler 4784 | BM; COI; K | 14.9 | -9.31667 |
| *Khaya anthoteca* | CUANZA NORTE | Golungo Alto. Serra do Alto Queta | Welwitsch 1314 | LISU; B; BM; COI; K | 14.76667 | -9.26667 |
| *Khaya anthoteca* | CUANZA NORTE | Mussengue | Welwitsch 317 col. carp. | BM | 15.48333 | -9.28333 |
| *Khaya anthoteca* | CUANZA NORTE | Mussengue | Welwitsch 318 col. carp. | BM | 15.48333 | -9.28333 |
| *Khaya anthoteca* | MALANGE | Bondo e Bângala, Quela | Nolde 309 | B | 17.11667 | -9.2 |
| *Khaya anthoteca* | MALANGE | Ao pé da catarata do Duque de Bragança | Dechamps, Murta & M.Silva 1492 | LISC | 16.05 | -9.1 |
| *Khaya ivorensis* | CABINDA | Bôma-Lubinda | Moura 476 | LISC | 12.13333 | -5.03333 |
| *Khaya ivorensis* | CABINDA | Bôma-Lubinda (Cacongo) | A. Silva 850 | LISC | 12.13333 | -5.21667 |
| *Khaya ivorensis* | CABINDA | Bôma-Lubinda (Cacongo) | A. Silva 851 | LISC | 12.13333 | -5.21667 |
| *Khaya ivorensis* | CABINDA | Bôma-Lubinda (Cacongo) | A. Silva 852 | LISC | 12.13333 | -5.21667 |
| *Khaya ivorensis* | CABINDA | Bôma-Lubinda (Cacongo) | A. Silva 853 | LISC | 12.13333 | -5.21667 |
| *Khaya ivorensis* | CABINDA | Bôma-Lubinda (Cacongo) | A. Silva 854 | LISC | 12.13333 | -5.21667 |
| *Khaya ivorensis* | CABINDA | Bôma-Lubinda (Cacongo) | A. Silva 855 | LISC | 12.13333 | -5.21667 |
| *Khaya ivorensis* | CABINDA | Bôma-Lubinda (Cacongo) | A. Silva 856 | LISC | 12.13333 | -5.21667 |
| *Khaya ivorensis* | CABINDA | Bôma-Lubinda (Cacongo) | A. Silva 857 | LISC | 12.13333 | -5.21667 |
| *Khaya ivorensis* | CABINDA | Bôma-Lubinda (Dinge) | A. Silva 480 | LISC | 12.36667 | -4.96667 |
| *Khaya ivorensis* | CABINDA | Bôma-Lubinda (Dinge) | A. Silva 485 | LISC | 12.36667 | -4.96667 |
| *Khaya ivorensis* | CABINDA | Bôma-Lubinda (Dinge) | A. Silva 486 | LISC | 12.36667 | -4.96667 |
| *Khaya ivorensis* | CABINDA | Bôma-Lubinda (Dinge) | A. Silva 492 | LISC | 12.36667 | -4.96667 |
| *Khaya ivorensis* | CABINDA | Bôma-Lubinda (Dinge) | A. Silva 500 | LISC | 12.36667 | -4.96667 |
| *Khaya ivorensis* | CABINDA | Bôma-Lubinda (Dinge) | A. Silva 503 | LISC | 12.36667 | -4.96667 |
| *Khaya ivorensis* | CABINDA | Maiombe, Pango Mongo, margens do rio Luango | Gossweiler 6252 | BM; COI; LISU; LISC | 12.5 | -5.06667 |
| *Khaya ivorensis* | CABINDA | Maiombe, Pango Mongo, margens do rio Luango | Gossweiler 6281 | BM; COI; K; LISC; LISU | 12.5 | -5.06667 |
| *Khaya ivorensis* | CABINDA | Maiombe, Chiaca | Cameira 124 | LISC | 12.53333 | -4.95 |
| *Khaya ivorensis* | CABINDA | Maiombe, Chiaca | Cameira 233 | LISC | 12.53333 | -4.95 |
| *Milicia excelsa* | CABINDA | Maiombe, Buco Zau | Gossweiler 6737 | LISU; LISC | 12.55 | -4.76667 |
| *Milicia excelsa* | CABINDA | Maiombe, Necuto | Gossweiler 6805 | LISU; LISC | 12.61667 | -4.91667 |
| *Milicia excelsa* | CUANZA NORTE | Golungo Alto, perto de Songue! | Welwitsch 1559 | LISU | 14.66667 | -9.11667 |
| *Oxystigma oxyphyllum* | CABINDA | Maiombe, Pango Mongo | Gossweiler 6093 | BM; COI; K; LISU; LISC | 12.5 | -5.03333 |
| *Oxystigma oxyphyllum* | CABINDA | Maiombe, Pango Mongo | Gossweiler 6093b | BM; LISU | 12.5 | -5.03333 |
| *Oxystigma oxyphyllum* | CABINDA | Baixo Maiombe, Ncanda, Mbaco | Gossweiler 6093c | LISC | 12.48333 | -5.05 |
| *Oxystigma oxyphyllum* | CABINDA | Baixo Maiombe, Ncanda, Mbaco | Gossweiler 6093e | LISC | 12.48333 | -5.05 |
| *Oxystigma oxyphyllum* | CABINDA | Belize | Gossweiler 7135 | K; LISU | 12.65 | -4.58333 |
| *Oxystigma oxyphyllum* | CABINDA | Belize, rio Luali | Gossweiler 7136 | BM; COI; K; LISU; LISC | 12.65 | -4.65 |
| *Oxystigma oxyphyllum* | CABINDA | Maiombe, Buco Zau (Chiaca) | MEFA -Semedo 425 | LISC | 12.53333 | -4.95 |
| *Oxystigma oxyphyllum* | CABINDA | Maiombe, Buco Zau (Chiaca) | MEFA -D´Orey 811 | LISC | 12.53333 | -4.95 |
| *Oxystigma oxyphyllum* | CABINDA | Dinge. Macanga, estrada de Massabi | Pinto 448 | COI; LISC | 12.08333 | -4.96667 |
| *Pterocarpus angolensis* | BENGUELA | Ganda | Andrada 92 | LISC; LISU | 14.66667 | -13.03333 |
| *Pterocarpus angolensis* | BENGUELA | Ganda, Chiqueque | V.Almeida 747 | LISC | 14.66667 | -13.03333 |
| *Pterocarpus angolensis* | BENGUELA | Ganda | Teixeira & Andrade, 7183 | LISC | 14.66667 | -13.03333 |
| *Pterocarpus angolensis* | BENGUELA | Chongoroi, Mapupa, margens do rio Chongoroi | Henriques 1210 | LISC; LISU | 13.8 | -13.35 |
| *Pterocarpus angolensis* | BENGUELA | Cubal | Lains & Silva 4 | LISC | 14.05 | -12.43333 |
| *Pterocarpus angolensis* | BIÉ | Cuemba, picada para Mussengue, na margem rio Cuíva | R.Santos 1739 | COI; LISC | 18.05 | -12.23333 |
| *Pterocarpus angolensis* | BIÉ | Nhárêa | R.Monteiro & Murta 2051 | COI; LISC | 17.05 | -11.41667 |
| *Pterocarpus angolensis* | BIÉ | Cuemba, picada para o Mussengue | R.Monteiro & Murta 1572 | COI; LISC | 18.05 | -12.23333 |
| *Pterocarpus angolensis* | BIÉ | Cuito, Bela Vista km 8 | Bamps, S. Martins & Maia 4131 | LISC | 16.9 | -12.45 |
| *Pterocarpus angolensis* | BIÉ | Tunda | Mazzochi-Alemanni 104 | K | 16.75 | -12.08333 |
| *Pterocarpus angolensis* | CUANDO-CUBANGO | Menongue, Cutato | Mendes 2005 | LISC | 16.6 | -14.55 |
| *Pterocarpus angolensis* | CUANDO-CUBANGO | Caiundo, Mato perto da Missão Capico | Mendes 2298 | LISC | 17.63333 | -15.38333 |
| *Pterocarpus angolensis* | CUANDO-CUBANGO | Cuito-Cuanavale | Mendes 2808 | LISC | 19.21667 | -15.15 |
| *Pterocarpus angolensis* | CUANDO-CUBANGO | Menongue, margens do rio Cuebe | P. Almeida 383 | LISC | 17.68333 | -14.7 |
| *Pterocarpus angolensis* | CUANDO-CUBANGO | Cuito-Cuanavale, prox. dos rios Cuito e Cuanavale | Teixeira 37 | BM; LISC | 19.21667 | -15.13333 |
| *Pterocarpus angolensis* | CUANDO-CUBANGO | Cuangar, entre Mucusso e posto administrativo e Luiana | R. Santos 2471 | LISC | 22.2 | -17.55 |
| *Pterocarpus angolensis* | CUANZA NORTE | Moxito do Luinga. Camabatela | Teixeira & al. 12061 | COI; LISC | 15.58333 | -8.4 |
| *Pterocarpus angolensis* | CUANZA SUL | Colonato da Cela | Teixeira & Sales 6078 | LISC | 15.11667 | -11.41667 |
| *Pterocarpus angolensis* | CUANZA SUL | Colonato da Cela | Teixeira & Sales 6224 | LISC | 15.11667 | -11.41667 |
| *Pterocarpus angolensis* | CUANZA SUL | Mata de Panda, Colonato de Cela | Teixeira & Figueira 5894 | LISC | 15.11667 | -11.41667 |
| *Pterocarpus angolensis* | CUANZA SUL | Colonato de Cela | Teixeira & Figueira 5934 | LISC | 15.11667 | -11.41667 |
| *Pterocarpus angolensis* | CUANZA SUL | N´Gunza, Quibala | R.Santos 1247 | COI; LISC | 14.98333 | -10.76667 |
| *Pterocarpus angolensis* | CUNENE | Cuamato, Mucope, Chica, Pocolo | Henriques 246 | LISC; LISU | 15.16667 | -16.33333 |
| *Pterocarpus angolensis* | CUNENE | Alto Cunene, Quipungo | J.J.Silva s.n. | P | 14.56667 | -14.81667 |
| *Pterocarpus angolensis* | CUNENE | Baixo Cunene, Namacunde, prox. de Ondjiva | Powell-Cotton col carp. 1524 | BM | 15.85 | -17.3 |
| *Pterocarpus angolensis* | CUNENE | Baixo Cunene, Chiede | Teixeira 3129 | LISC | 16 | -17.16667 |
| *Pterocarpus angolensis* | CUNENE | Baixo Cunene, Chiede | Menezes 974 | LISC | 16 | -17.16667 |
| *Pterocarpus angolensis* | CUNENE | Xangongo, ao km 50,5 do trajecto Cuamato-Ondjiva | Menezes 3171 | LISC | 15.58333 | -17.08333 |
| *Pterocarpus angolensis* | HUAMBO | Fazenda Aurora | Oliveira 32 | COI | 15.96667 | -11.96667 |
| *Pterocarpus angolensis* | HUAMBO | Bailundo, Chitende | Andrada 6 | LISC | 15.93333 | -12.16667 |
| *Pterocarpus angolensis* | HUAMBO | Caála, Lepi | Lynes s.n. | BM | 15.43333 | -12.86667 |
| *Pterocarpus angolensis* | HUAMBO | Huambo, Chianga | Teixeira & Sousa 6793 | COI; LISC | 15.48333 | -12.88333 |
| *Pterocarpus angolensis* | HUAMBO | Planalto do Huambo | Gossweiler 51 | LISC | 15.78333 | -12.73333 |
| *Pterocarpus angolensis* | HUAMBO | Huambo, Centro de estudos da Chianga | M. Silva 2844 | COI; LISC | 15.48333 | -12.88333 |
| *Pterocarpus angolensis* | HUAMBO | Estação IIAA | Dechamps, Murta & M. Silva 1013 | LISC | 15.78333 | -12.78333 |
| *Pterocarpus angolensis* | HUÍLA | Caconda, entre Chicomba e Quipungo | Powell-Cotton 85 | BM | 14.91667 | -14.33333 |
| *Pterocarpus angolensis* | HUÍLA | Quilengues, Impulo | R. Santos 259 | LISC | 13.65 | -13.88333 |
| *Pterocarpus angolensis* | HUÍLA | Quilengues, Bonga | Teixeira & Andrade, 4331 | COI; LISC | 13.96667 | -14.26667 |
| *Pterocarpus angolensis* | HUÍLA | Huíla | Antunes 177 | LISC | 13.53333 | -15.06667 |
| *Pterocarpus angolensis* | HUÍLA | Huíla | Antunes 625 | LISC | 13.53333 | -15.06667 |
| *Pterocarpus angolensis* | HUÍLA | Huíla | Antunes 629 | P | 13.53333 | -15.06667 |
| *Pterocarpus angolensis* | HUÍLA | Lubango, Chivinguiro, entre a Missão e a lagoa de Ontite | Correia 1298 | LISC | 13.53333 | -15.06667 |
| *Pterocarpus angolensis* | HUÍLA | Huíla | Dekindt s.n. | LISC | 13.53333 | -15.06667 |
| *Pterocarpus angolensis* | HUÍLA | Chibia, do Jau para Chivinguiro | Mendes 1560 | LISC | 13.41667 | -15.16667 |
| *Pterocarpus angolensis* | HUÍLA | Chibia, do Jau para Chivinguiro | Mendes 1561 | LISC | 13.41667 | -15.16667 |
| *Pterocarpus angolensis* | HUÍLA | Lubango, Humpata | A.S.Monteiro 11 | COI | 13.36667 | -15 |
| *Pterocarpus angolensis* | HUÍLA | Huíla | Teixeira 1701 | BR; LISC | 13.53333 | -15.06667 |
| *Pterocarpus angolensis* | HUÍLA | Lubango, Humpata, Jau | Teixeira 3349 | LISC | 13.5 | -15.15 |
| *Pterocarpus angolensis* | HUÍLA | Chibia, Km 17 da estrada da Chibia | Teixeira & Figueira 3915 | COI; LISC | 13.91667 | -15.3 |
| *Pterocarpus angolensis* | HUÍLA | Lubango, Lopolo, rio Munhino | Welwitsch 1863 | BR; LISU; COI | 13.55 | -15.01667 |
| *Pterocarpus angolensis* | HUÍLA | Lubango, Lopolo | Welwitsch col. carp. 440 | BM | 13.5 | -15.06667 |
| *Pterocarpus angolensis* | HUÍLA | Lubango, Lopolo | Welwitsch col. carp. 441 | BM | 13.5 | -15.06667 |
| *Pterocarpus angolensis* | HUÍLA | Ganguelas, ao km 1 da bifurcação Cubango-Jamba | Couto 273 | LISC | 16 | -14.5 |
| *Pterocarpus angolensis* | HUÍLA | Alto Cunene, na picada Mulondo-Capelongo, ao km 81 | Menezes 862 | LISC | 15.3 | -15.08333 |
| *Pterocarpus angolensis* | HUÍLA | Chibia, Jau, Bata-bata | Menezes 2358 | LISC | 13.4 | -15.25 |
| *Pterocarpus angolensis* | HUÍLA | Lubango, Chibia, a 4km para Jau | R. Santos 626 | LISC | 13.65 | -15.16667 |
| *Pterocarpus angolensis* | HUÍLA | Quilengues, Impulo | R: Santos 259 | LISC | 13.63333 | -13.86667 |
| *Pterocarpus angolensis* | LUNDA NORTE | Chitato, Dundo, rio Luachimo | Gossweiler 13733 | BM; K; P | 20.83333 | -7.35 |
| *Pterocarpus angolensis* | LUNDA NORTE | Minungo, Cacolo | Young 704 | BM; BR | 19.28333 | -10.11667 |
| *Pterocarpus angolensis* | LUNDA NORTE | Dundo | Companhia dos Diamantes 108 | LISC | 20.78333 | -7.33333 |
| *Pterocarpus angolensis* | LUNDA SUL | Saurimo, | Carrisso & Mendonça 506 | BM; COI | 20.25 | -9.5 |
| *Pterocarpus angolensis* | LUNDA SUL | Saurimo sem localidade precisa | Lynes 270 | BR | 20.25 | -9.5 |
| *Pterocarpus angolensis* | LUNDA SUL | Saurimo sem localidade precisa | Lynes 343 | BR | 20.25 | -9.5 |
| *Pterocarpus angolensis* | LUNDA SUL | Chitato, estrada do Dundo, Sombo, Km 85 | Luna de Carvalho Veg. 33 | LISC | 20.95 | -7.5 |
| *Pterocarpus angolensis* | MALANGE | Nas matas de Malange | Marques 45 | COI | 16.16667 | -9.83333 |
| *Pterocarpus angolensis* | MALANGE | Cacuso, Quizenza | Gossweiler 1387 | K; P | 15.45 | -9.35 |
| *Pterocarpus angolensis* | MALANGE | Pungo Andongo | Gossweiler 5297 | BM; COI; LISC; LISU | 15.58333 | -9.66667 |
| *Pterocarpus angolensis* | MALANGE | Cacuso, de Zamba e Pungo Andongo, margens rio Lutete | Welwitsch 1864 | BM; COI; LISU | 15.75 | -9.46667 |
| *Pterocarpus angolensis* | MALANGE | Entre Quela e Belo Horizonte | Marques 115 | COI; LISU | 17 | -9 |
| *Pterocarpus angolensis* | MALANGE | Capunda, Mulundo (Reserva da Palanca Preta Gigante) | Menezes 1922 | LISC | 17.66667 | -11.41667 |
| *Pterocarpus angolensis* | MALANGE | Malange | C.T.Almeida s.n. | LISC | 16.33333 | -9.53333 |
| *Pterocarpus angolensis* | MALANGE | Malange sem localidade presisa | Gossweiler 1372 | BM; K | 16.33333 | -9.53333 |
| *Pterocarpus angolensis* | MOXICO | Luena, Polígono Florestal, prox. do rio Luena | Càmeira 11 | LISC | 19.9 | -11.8 |
| *Pterocarpus angolensis* | MOXICO | Luena | R.Monteiro, R.Santos & Murta 487 | LISC | 19.9 | -11.76667 |
| *Pterocarpus angolensis* | MOXICO | Luau, Cafungo | Soares s.n. | LISC | 22.11667 | -10.91667 |
| *Pterocarpus angolensis* | MOXICO | Estrada Luena-Saurimo a 12km de Luena | Barbosa 11077 | LISC | 19.91667 | -11.66667 |
| *Pterocarpus angolensis* | MOXICO | Estrada Luena-Saurimo a 12km de Luena | Barbosa 11065 | COI; LISC | 19.91667 | -11.66667 |
| *Pterocarpus angolensis* | MOXICO | Luena. Centro de estudos | Teixeira & Pedro 7599 | COI; LISC | 19.9 | -11.76667 |
| *Pterocarpus angolensis* | NAMIBE | Bibala | Gossweiler 1764 | LISC | 13.26667 | -14.76667 |
| *Pterocarpus angolensis* | NAMIBE | Bibala | Gossweiler 12764 | LISC | 13.26667 | -14.76667 |
| *Pterocarpus angolensis* | NAMIBE | Bibala, Catchundo | Teixeira 2400 | BR | 13.26667 | -14.76667 |
| *Pterocarpus angolensis* | UIGE | Entre a ponte do Zadi e Béu | Raimundo, Matos & Figueira 471 | LISC | 15.36667 | -6.2 |
| *Pterocarpus angolensis* | UIGE | Entre Buenga Sul e (Buenga Norte), andados 5km | Raimundo, Matos & Figueira 908 | LISC | 15.88333 | -6.96667 |
| *Pterocarpus tinctorius* | BENGO | Dande, Cassalengues | Araújo 96 | COI; LISC | 13.9 | -8.48333 |
| *Pterocarpus tinctorius* | BENGO | Ambriz, Quibocolo | R.Monteiro, R.Santos & Murta 431 | COI; LISC | 13.1 | -7.83333 |
| *Pterocarpus tinctorius* | BENGO | Libongo, rio Lifune | Welwitsch 1870 | BM; COI; LISU | 13.48333 | -8.38333 |
| *Pterocarpus tinctorius* | BENGO | Libongo, rio Lifune | Welwitsch col. carp. 438 | BM | 13.48333 | -8.38333 |
| *Pterocarpus tinctorius* | BENGO | Luanda- Caxito, Ucua | R.Monteiro, R.Santos & Murta 184 | LISC | 14.16667 | -8.65 |
| *Pterocarpus tinctorius* | CUANZA NORTE | Estrada Camabatela-Cangola, Mata Panda | Teixeira & al. 12221 | COI; LISC | 15.6 | -8.08333 |
| *Pterocarpus tinctorius* | CUANZA NORTE | Cazengo, Granja de S. Luís: Estação agrícola de Cazengo | Gossweiler 5437 | BM; COI; LISC | 14.9 | -9.31667 |
| *Pterocarpus tinctorius* | CUANZA NORTE | Cazengo, Granja, Estação agrícola de Cazengo de S. Luís | Gossweiler 5518 | BM; COI; LISC | 14.9 | -9.31667 |
| *Pterocarpus tinctorius* | CUANZA NORTE | Cazengo, Granja de S. Luís, Estação agrícola de Cazengo | Gossweiler 5915 | BM; COI; K; LISC; LISU | 14.9 | -9.31667 |
| *Pterocarpus tinctorius* | CUANZA NORTE | Cazengo, Granja de S. Luís, Estação agrícola de Cazengo | Gossweiler 5915b | LISC | 14.9 | -9.31667 |
| *Pterocarpus tinctorius* | CUANZA NORTE | Cazengo, Granja de S. Luís, Estação agrícola de Cazengo | Gossweiler 5915c | LISC | 14.9 | -9.31667 |
| *Pterocarpus tinctorius* | CUANZA NORTE | Dembos, Castende, prox. do rio Zenza | Gossweiler 8448 | BM | 14.26667 | -8.83333 |
| *Pterocarpus tinctorius* | CUANZA NORTE | Dembos, Úcua, Cacundo e rio Dande | R. Monteiro, R.Santos & Murta 341 | COI; LISC | 14 | -8.5 |
| *Pterocarpus tinctorius* | CUANZA NORTE | Zenza do Golungo, entre Calunguembo e Tanderachique | Welwitsch 1866 | BM; LISU | 13.95 | -9.03333 |
| *Pterocarpus tinctorius* | CUANZA NORTE | Entre Calunguembo e Calolo | Welwitsch col. carp. 437 | BM | 14.96667 | -9.21667 |
| *Pterocarpus tinctorius* | CUANZA NORTE | Golungo Alto (prox. de Cabanga-Caculungo) | Welwitsch 1867 | BM; COI; LISU | 14.78333 | -9.13333 |
| *Pterocarpus tinctorius* | CUANZA NORTE | De Calolo a Trombeta, Tchamba até Sange | Welwitsch 1867b | BM; LISU | 14.61667 | -9.18333 |
| *Pterocarpus tinctorius* | CUANZA NORTE | Serra do Alto Queta | Welwitsch 1868 | BM; COI; LISU | 14.76667 | -9.26667 |
| *Pterocarpus tinctorius* | CUANZA NORTE | Cazengo, Serra de Muxaúla | Welwitsch 1869 | BM; COI; LISU | 15 | -9.25 |
| *Pterocarpus tinctorius* | CUANZA NORTE | Na estrada para Golungo Alto a 7km do Zenza | Raimundo, Matos & Figueira 194 | LISC | 14.25 | -9.21667 |
| *Pterocarpus tinctorius* | CUANZA SUL | Entre Quissongo e Haco andados 28km | Raimundo, Matos & Figueira 691 | LISC | 15.28333 | -10.2 |
| *Pterocarpus tinctorius* | CUANZA SUL | A cerca de 15km do rio Cuanza, picada para Quissaquina | Raimundo, Matos & Figueira 695 | LISC | 15.26667 | -9.68333 |
| *Pterocarpus tinctorius* | CUANZA SUL | Junto da ponte da Cachoeira, 40km de N´Gunza | Teixeira & al. 11257 | LISC | 14.1 | -10.98333 |
| *Pterocarpus tinctorius* | CUANZA SUL | Calulo | Barbosa 11152 | COI; LISC | 14.86667 | -10 |
| *Pterocarpus tinctorius* | CUANZA SUL | Entre Quibala e Muquitixe | Lopes, M. Silva & Murta 3727 | COI; LISC | 15 | -10.5 |
| *Pterocarpus tinctorius* | CUANZA SUL | Libolo | Dawe 368 | K | 14.76667 | -10.11667 |
| *Pterocarpus tinctorius* | CUANZA SUL | Quissama | Geraldes s.n. | LISC | 14.48333 | -9.98333 |
| *Pterocarpus tinctorius* | CUANZA SUL | Amboim, prox de Carlaongo-Cuvo | Gossweiler 9877 | BR; COI; K; LISC | 13.88333 | -10.86667 |
| *Pterocarpus tinctorius* | CUANZA SUL | Amboim, prox de Carlaongo-Cuvo | Gossweiler 9877b | BM; LISC | 13.88333 | -10.86667 |
| *Pterocarpus tinctorius* | CUANZA SUL | Quibala, ao km 33 da estrada Dondo-Quibala | Menezes 3373 | LISC | 14.9 | -10.45 |
| *Pterocarpus tinctorius* | LUANDA | Luanda, Vila Oledo, rio Dande | R.Monteiro, R.Santos & Murta 188 | LISC | 13.63333 | -8.61667 |
| *Pterocarpus tinctorius* | LUANDA | Luanda, Vila Oledo, rio Dande | R.Monteiro, R.Santos & Murta 193 | COI | 13.63333 | -8.61667 |
| *Pterocarpus tinctorius* | LUANDA | Luanda | Gossweiler 13955 | LISC | 13.23333 | -8.8 |
| *Pterocarpus tinctorius* | LUANDA | Luanda | Gossweiler 13955b | COI; LISC | 13.23333 | -8.8 |
| *Pterocarpus tinctorius* | LUANDA | Viana. Vale do Bengo, picada Q | Teixeira 10203 | LISC | 13.36667 | -8.91667 |
| *Pterocarpus tinctorius* | MALANGE | Entre Cacuso e Pungo Andongo a 13km de Cacuso | Barbosa 11329 | COI; LISC | 15.75 | -9.5 |
| *Pterocarpus tinctorius* | MALANGE | Cacuso, Pungo Andongo | Gossweiler 5293 | BM; COI; LISC | 15.58333 | -9.66667 |
| *Pterocarpus tinctorius* | MALANGE | Bondo e Bângala, Baixa de Cassanje, rio Lui | Gossweiler 9521 | BM; COI; K; LISC | 17.5 | -9.05 |
| *Pterocarpus tinctorius* | MALANGE | Bondo e Bângala, Quela | Nolde 195 | BM | 17.11667 | -9.2 |
| *Pterocarpus tinctorius* | MALANGE | Capunda, ao km 32 do trajecto (Cachoeiras do Dando) | Menezes 2159 | LISC | 17.5 | -10.78333 |
| *Pterocarpus tinctorius* | MALANGE | A 4 km de Cacuso vers Pungo Andongo | Dechamps, Murta & M.Silva 1507 | LISC | 15.81667 | -9.5 |
| *Pterocarpus tinctorius* | MALANGE | A 4 km de Cacuso vers Pungo Andongo | Dechamps, Murta & M.Silva 1508 | LISC | 15.81667 | -9.5 |
| *Pterocarpus tinctorius* | MALANGE | A 4 km de Cacuso vers Pungo Andongo | Dechamps, Murta & M.Silva 1509 | LISC | 15.81667 | -9.5 |
| *Pterocarpus tinctorius* | MALANGE | A 11 km de Cacuso vers Pungo Andongo | Dechamps, Murta & M.Silva 1510 | LISC | 16.83333 | -9.55 |
| *Pterocarpus tinctorius* | MALANJE | Pungo Andongo (Sobado, Cabanga-Quiqueta) | Welwitsch 1871 | BM; COI; LISU | 15.58333 | -9.66667 |
| *Pterocarpus tinctorius* | MALANJE | Entre Lombe e Quibinda | Welwitsch 1872 | BM; LISU | 16.21667 | -9.61667 |
| *Pterocarpus tinctorius* | ZAIRE | Soyo, Lunuango, prox. de Ganga-Ginga | Dawe 34 | K | 12.61667 | -6.5 |
| *Pterocarpus tinctorius* | ZAIRE | Junto à ponte do rio M`Pozo na estrada para Noqui | Raimundo, Matos & Figueira 801 | LISC | 13.86667 | -5.98333 |
| *Pterocarpus tinctorius* | ZAIRE | Congo, sem localidade precisa | Dawe 58 | K | 14.7 | -6.26667 |
| *Terminalia superba* | CABINDA | Bôma-Lubinda (Cacongo) | A. Silva 485 | LISC | 12.13333 | -5.21667 |
| *Terminalia superba* | CABINDA | Bôma-Lubinda (Cacongo) | A. Silva 486 | LISC | 12.13333 | -5.21667 |
| *Terminalia superba* | CABINDA | Bôma-Lubinda (Cacongo) | A. Silva 490 | COI; LISC | 12.13333 | -5.21667 |
| *Terminalia superba* | CABINDA | Bôma-Lubinda (Cacongo) | A. Silva 878 | LISC | 12.13333 | -5.21667 |
| *Terminalia superba* | CABINDA | Bôma-Lubinda (Cacongo) | A. Silva 879 | COI; LISC | 12.13333 | -5.21667 |
| *Terminalia superba* | CABINDA | Bôma-Lubinda (Dinge) | Pinto 463 | LISC | 12.36667 | -4.96667 |
| *Terminalia superba* | CABINDA | Bôma-Lubinda (Dinge) | A. Silva 481 | LISC | 12.36667 | -4.96667 |
| *Terminalia superba* | CABINDA | Bôma-Lubinda (Dinge) | A. Silva 482 | LISC | 12.36667 | -4.96667 |
| *Terminalia superba* | CABINDA | Bôma-Lubinda (Dinge) | A. Silva 487 | COI; LISC | 12.36667 | -4.96667 |
| *Terminalia superba* | CABINDA | Bôma-Lubinda (Dinge) | A. Silva 489 | COI; LISC | 12.36667 | -4.96667 |
| *Terminalia superba* | CABINDA | Bôma-Lubinda (Dinge) | A. Silva 493 | LISC | 12.36667 | -4.96667 |
| *Terminalia superba* | CABINDA | Bôma-Lubinda (Dinge) | A. Silva 494 | LISC | 12.36667 | -4.96667 |
| *Terminalia superba* | CABINDA | Bôma-Lubinda (Dinge) | A. Silva 497 | COI; LISC | 12.36667 | -4.96667 |
| *Terminalia superba* | CABINDA | Bôma-Lubinda (Dinge) | A. Silva 502 | COI; LISC | 12.36667 | -4.96667 |
| *Terminalia superba* | CABINDA | Maiombe, Chiaca, Buco Zau | R.Monteiro, R.Santos & Murta 319 | LISC | 12.53333 | -4.95 |
| *Terminalia superba* | CABINDA | Maiombe, Chiaca, Buco Zau | R.Monteiro, R.Santos & Murta 368 | LISC | 12.53333 | -4.95 |
| *Terminalia superba* | CABINDA | Maiombe, margens do rio Chiaca, Buco Zau | Murta 44 | COI; LISC; LISU | 12.53333 | -4.95 |
| *Terminalia superba* | CABINDA | Pango Mongo, margens do rio Loango | Gossweiler 6218 | BM; COI; LISC; LISU | 12.5 | -5.03333 |
| *Terminalia superba* | CABINDA | Maiombe, Chiaca, Buco Zau | MEFA- Carvalho 326 | LISC | 12.53333 | -4.95 |
| *Terminalia superba* | CABINDA | Maiombe, Chiaca, Buco Zau | MEFA- Semedo 533 | LISC | 12.53333 | -4.95 |
| *Terminalia superba* | CABINDA | Maiombe | MEFA- D´Orey 826 | LISC | 12.58333 | -4.73333 |
| *Terminalia superba* | CABINDA | Belize | MEFA- D´Orey 743 | LISC | 12.65 | -4.58333 |
| *Terminalia superba* | CABINDA | Pango Mongo, margens do rio Loango | Gossweiler 6218b | BM; LISC | 12.5 | -5.03333 |
| *Terminalia superba* | CABINDA | Pango Mongo, margens do rio Loango, Baixo Maiombe | Gossweiler 6120 | BM; COI; LISC; LISU | 12.5 | -5.03333 |

*LISC (Tropical Research Institute, Lisbon), LISU (University of Lisbon), COI (University of Coimbra), BM (Natural History Museum, London) and K (Royal Botanic Gardens Kew). Specimens housed in B (Botanischer Garten und Botanisches Museum Berlin-Dahlem), BR (National Botanic Garden of Belgium) and P (Muséum National d'Histoire Naturelle) herbaria were georeferenced using the collecting site description referred in Conspectus Florae Angolensis [37, 39, 40, 41].
