# Supplementary material for: Documenting Biogeographical Patterns of African Timber Species Using Herbarium Records: A Conservation Perspective Based on Native Trees from Angola
Source: PLoS One. 2014 Jul 25;9(7):e103403. doi: 10.1371/journal.pone.0103403 (PMC4111583; doi:10.1371/journal.pone.0103403)
Supplement: Table S2 — Sixty-two datasets from GBIF providers queried for the 18 timber species, producing 2253 records. Accessed 29 April 2013. (DOC) [file pone.0103403.s004.doc]

**Table S2.** Sixty-two datasets from GBIF providers queried for the 18 timber species, producing 2253 records. Accessed 29 April 2013

| **Dataset** | **Institution code** | **Data Publisher** | **Accessed through GBIF data portal** | **Publisher Country** |
| --- | --- | --- | --- | --- |
| Base de datos para la xiloteca del Instituto de Biología de la UNAM | Tw | Comisión nacional para el conocimiento y uso de la biodiversidad | http://data.gbif.org/datasets/resource/13386 | Mexico |
| Bishop Museum Natural Sciences Data | BPBM | Bernice Pauahi Bishop Museum | http://data.gbif.org/datasets/resource/54 | United States |
| Botanic Garden of Finnish Museum of Natural History | H | Finnish Museum of Natural History | http://data.gbif.org/datasets/resource/2406 | Finland |
| Botanical Museum, Copenhagen. Database of type specimens | C | Botanical Garden & Museum, Natural History Museum of Denmark | http://data.gbif.org/datasets/resource/716 | Denmark |
| Botany (UPS) | UPS | GBIF-Sweden | http://data.gbif.org/datasets/resource/1045 | Sweden |
| Cameroon National Herbarium | YA | Cameroon National Herbarium | http://data.gbif.org/datasets/resource/1474 | Cameroon |
| Computarización de la xiloteca Dr. Faustino Miranda del Instituto de Ecología, AC | No proporcionado | Comisión nacional para el conocimiento y uso de la biodiversidad | http://data.gbif.org/datasets/resource/13104 | Mexico |
| CONN | UConn | University of Connecticut | http://data.gbif.org/datasets/resource/13544 | United States |
| Database Schema for UC Davis [Herbarium Labels] | UCD | University of California, Davis | http://data.gbif.org/datasets/resource/734 | United States |
| EURISCO, The European Genetic Resources Search Catalogue | GBR004 | Bioversity International | http://data.gbif.org/datasets/resource/1905 | International |
| Fairchild Tropical Botanic Garden Virtual Herbarium Darwin Core format | FTG | Fairchild Tropical Botanic Garden | http://data.gbif.org/datasets/resource/202 | United States |
| Field Museum of Natural History (Botany) Seed Plant Collection | F | Field Museum | http://data.gbif.org/datasets/resource/14346 | United States |
| Harvard University Herbaria | Harvard University | Harvard University Herbaria | http://data.gbif.org/datasets/resource/1827 | United States |
| HBGSpermatophyta - Herbarium Hamburgense | HBG | Herbarium Hamburgense | http://data.gbif.org/datasets/resource/1604 | Germany |
| Herbaria of the University and ETH Zürich | Z | Herbaria of the University and ETH Zürich (Z+ZT) | http://data.gbif.org/datasets/resource/11516 | Switzerland |
| Herbarium Berolinense | BGBM | Botanic Garden and Botanical Museum Berlin-Dahlem | http://data.gbif.org/datasets/resource/1095 | Germany |
| Herbarium of The New York Botanical Garden | NY | The New York Botanical Garden | http://data.gbif.org/datasets/resource/8967 | United States |
| Herbarium of the Université Libre de Bruxelles | ULB | BeBIF Provider | http://data.gbif.org/datasets/resource/9102 | Belgium |
| Herbarium Senckenbergianum (FR) | FR | Senckenberg | http://data.gbif.org/datasets/resource/8311 | Germany |
| Herbarium Specimen of the Institute of Traditional Medicine, Tanzania | ITMH | TanBIF | http://data.gbif.org/datasets/resource/13540 | Tanzania |
| Herbarium specimens | UM2 | Herbarium of Université de Montpellier 2, Institut de Botanique | http://data.gbif.org/datasets/resource/13688 | France |
| Herbarium togoense | Herbarium togoense | Université de Lomé | http://data.gbif.org/datasets/resource/12767 | Togo |
| Herbarium WU | WU | University of Vienna, Institute for Botany - Herbarium WU | http://data.gbif.org/datasets/resource/1496 | Austria |
| Herbier du Bénin | HNB | Université d'Abomey-Calavi, Faculté des Sciences Agronomiques | http://data.gbif.org/datasets/resource/13459 | Benin |
| Herbier Ecole de Faune | EFG | Ecole de Faune de Garoua | http://data.gbif.org/datasets/resource/12796 | Cameroon |
| IICT Herbário LISC | IICT | Instituto de Investigação Científica Tropical | http://data.gbif.org/datasets/resource/12700 | Portugal |
| Jardín Botánico de Córdoba: Herbarium COA | COA | GBIF-Spain | http://data.gbif.org/datasets/resource/247 | Spain |
| Millenium Seedbank (MSB) | SANBI | South African National Biodiversity Institute | http://data.gbif.org/datasets/resource/12714 | South Africa |
| Missouri Botanical Garden | MO | Missouri Botanical Garden | http://data.gbif.org/datasets/resource/12084 | United States |
| Nationaal Herbarium Nederland | L | Netherlands Centre for Biodiversity Naturalis, section National Herbarium of the Netherlands | http://data.gbif.org/datasets/resource/11520 | Netherlands |
| Natural History Museum, Vienna - Herbarium W | W | Natural History Museum, Vienna - Herbarium W | http://data.gbif.org/datasets/resource/13042 | Austria |
| NMNH Botany Collections | US | National Museum of Natural History, Smithsonian Institution | http://data.gbif.org/datasets/resource/1874 | United States |
| Peabody Paleobotany DiGIR Service | YPM | Yale University Peabody Museum | http://data.gbif.org/datasets/resource/8141 | United States |
| Peabody Paleoportal DiGIR Service (PB) | YPM | Yale University Peabody Museum | http://data.gbif.org/datasets/resource/8176 | United States |
| Phanérogames recensés aux Monts Nimba | SSMN | Centre d'Observation de Surveillance et d'Information Environnementales (COSIE) | http://data.gbif.org/datasets/resource/13405 | Guinea |
| Phanerogamic Botanical Collections (S) | S | GBIF-Sweden | http://data.gbif.org/datasets/resource/8113 | Sweden |
| Phanerogams herbarium specimens | MNHN | MNHN - Museum national d'Histoire naturelle | http://data.gbif.org/datasets/resource/13686 | France |
| PRECIS | SANBI | South African National Biodiversity Institute | http://data.gbif.org/datasets/resource/12716 | South Africa |
| PRECIS (KwaZulu-Natal Herbarium) | SANBI | South African National Biodiversity Institute | http://data.gbif.org/datasets/resource/12715 | South Africa |
| Precis Plant Data | SANBI | South African National Biodiversity Institute | http://data.gbif.org/datasets/resource/13505 | South Africa |
| Rapid Assessment Program (RAP) Biodiversity Survey Database | Conservation International | Conservation International | http://data.gbif.org/datasets/resource/8076 | United States |
| RBGE Herbarium (E) | E | Royal Botanic Garden Edinburgh | http://data.gbif.org/datasets/resource/8402 | UK |
| RBGE Living Collections | E | Royal Botanic Garden Edinburgh | http://data.gbif.org/datasets/resource/9167 | UK |
| Real Jardin Botanico (Madrid), Vascular Plant Herbarium (MA) | MA | GBIF-Spain | http://data.gbif.org/datasets/resource/240 | Spain |
| Royal Botanic Gardens, Kew | K | Royal Botanic Gardens, Kew | http://data.gbif.org/datasets/resource/629 | UK |
| Royal Museum of Central Africa - Metafro-Infosys - Prelude | RMCA-Metafro-Infosys | BeBIF Provider | http://data.gbif.org/datasets/resource/96 | Belgium |
| Royal Museum of Central Africa - Metafro-Infosys - Xylarium | RMCA-Metafro-Infosys | BeBIF Provider | http://data.gbif.org/datasets/resource/14521 | Belgium |
| SINGER Coordinator | ITA303 | Bioversity International | http://data.gbif.org/datasets/resource/8349 | International |
| Southern Cape herbarium | GRBGT | South African National Biodiversity Institute | http://data.gbif.org/datasets/resource/11954 | South Africa |
| Staatliches Museum für Naturkunde Stuttgart, Herbarium | STU | Botanic Garden and Botanical Museum Berlin-Dahlem | http://data.gbif.org/datasets/resource/1100 | Germany |
| SysTax - Botanical Gardens | BAYRT | SysTax | http://data.gbif.org/datasets/resource/14438 | Germany |
| The AAU Herbarium Database | AAU | Herbarium of the University of Aarhus | http://data.gbif.org/datasets/resource/224 | Denmark |
| The System-wide Information Network for Genetic Resources (SINGER) | ETH013 | Bioversity International | http://data.gbif.org/datasets/resource/1430 | International |
| The Vascular Plant Collection at the Botanische Staatssammlung München | M | Staatliche Naturwissenschaftliche Sammlungen Bayerns | http://data.gbif.org/datasets/resource/11996 | Germany |
| UA Herbarium | ARIZ | University of Arizona Herbarium | http://data.gbif.org/datasets/resource/7900 | United States |
| United States National Plant Germplasm System Collection | USA047 | US National Plant Germplasm System | http://data.gbif.org/datasets/resource/1429 | United States |
| University of Copenhagen's Arboretum | KVL | Arboretum, University of Copenhagen | http://data.gbif.org/datasets/resource/702 | Denmark |
| University of Ghana - Ghana Herbarium | LEGON-GC | Ghana Biodiversity Information Facility (GhaBIF) | http://data.gbif.org/datasets/resource/14195 | Ghana |
| USDA PLANTS Database | USDA NRCS | USDA PLANTS | http://data.gbif.org/datasets/resource/1066 | United States |
| WAHerb | PERTH | Western Australian Herbarium | http://data.gbif.org/datasets/resource/13675 | Australia |
| West African Vegetation Database | FR | Senckenberg | http://data.gbif.org/datasets/resource/14161 | Germany |
| West-, Central- and East African Plants Databases | FR | Senckenberg | http://data.gbif.org/datasets/resource/14190 | Germany |
