# Supplementary material for: Documenting Biogeographical Patterns of African Timber Species Using Herbarium Records: A Conservation Perspective Based on Native Trees from Angola
Source: PLoS One. 2014 Jul 25;9(7):e103403. doi: 10.1371/journal.pone.0103403 (PMC4111583; doi:10.1371/journal.pone.0103403)
Supplement: Table S3 — Data provider per species accessed through GBIF data portal for each of the 18 timber species considered in this study. Accessed 29 April 2013. (DOC) [file pone.0103403.s005.doc]

**Table S3.** Data provider per species accessed through GBIF data portal for each of the 18 timber species considered in this study. Accessed 29 April 2013

| **Family, species** | **Data Publisher, accessed through GBIF data portal** |
| --- | --- |
| **Combretaceae** |  |
| ***Terminalia superba* Engl. & Diels** | Harvard University Herbaria, http://data.gbif.org/datasets/resource/1827  HBGSpermatophyta - Herbarium Hamburgense, http://data.gbif.org/datasets/resource/1604  Herbarium of the Université Libre de Bruxelles, http://data.gbif.org/datasets/resource/9102  Herbarium Senckenbergianum (FR), http://data.gbif.org/datasets/resource/8311  Herbarium WU, http://data.gbif.org/datasets/resource/1496  IICT Herbário LISC, http://data.gbif.org/datasets/resource/12700  Missouri Botanical Garden, http://data.gbif.org/datasets/resource/12084  Nationaal Herbarium Nederland, http://data.gbif.org/datasets/resource/11520  Natural History Museum, Vienna - Herbarium W, http://data.gbif.org/datasets/resource/13042  NMNH Botany Collections, http://data.gbif.org/datasets/resource/1874  Phanérogames recensés aux Monts Nimba, http://data.gbif.org/datasets/resource/13405  Phanerogamic Botanical Collections (S), http://data.gbif.org/datasets/resource/8113  Phanerogams herbarium specimens, http://data.gbif.org/datasets/resource/13686  Rapid Assessment Program (RAP) Biodiversity Survey Database, http://data.gbif.org/datasets/resource/8076  RBGE Herbarium (E), http://data.gbif.org/datasets/resource/8402  Real Jardin Botanico (Madrid), Vascular Plant Herbarium (MA), http://data.gbif.org/datasets/resource/240  Royal Botanic Gardens, Kew, http://data.gbif.org/datasets/resource/629  Royal Museum of Central Africa - Metafro-Infosys - Xylarium, http://data.gbif.org/datasets/resource/14521  The Vascular Plant Collection at the Botanische Staatssammlung München, http://data.gbif.org/datasets/resource/11996  United States National Plant Germplasm System Collection, http://data.gbif.org/datasets/resource/1429  University of Ghana - Ghana Herbarium, http://data.gbif.org/datasets/resource/14195  West-, Central- and East African Plants Databases, http://data.gbif.org/datasets/resource/14190 |
| **Ebenaceae** |  |
| ***Diospyros mespiliformis* Hochst. ex A. DC.** | Botany (UPS), http://data.gbif.org/datasets/resource/1045  CONN, http://data.gbif.org/datasets/resource/13544  Database Schema for UC Davis [Herbarium Labels], http://data.gbif.org/datasets/resource/734  EURISCO, The European Genetic Resources Search Catalogue, http://data.gbif.org/datasets/resource/1905  Fairchild Tropical Botanic Garden Virtual Herbarium Darwin Core format, http://data.gbif.org/datasets/resource/202  Harvard University Herbaria, http://data.gbif.org/datasets/resource/1827  Herbarium Berolinense, http://data.gbif.org/datasets/resource/1095  Herbarium of The New York Botanical Garden, http://data.gbif.org/datasets/resource/8967  Herbarium Senckenbergianum (FR), http://data.gbif.org/datasets/resource/8311  Herbarium Specimen of the Institute of Traditional Medicine, Tanzania, http://data.gbif.org/datasets/resource/13540  Herbarium specimens, http://data.gbif.org/datasets/resource/13688  Herbarium togoense, http://data.gbif.org/datasets/resource/12767  Herbarium WU, http://data.gbif.org/datasets/resource/1496  Herbier du Bénin, http://data.gbif.org/datasets/resource/13459  Herbier Ecole de Faune, http://data.gbif.org/datasets/resource/12796  IICT Herbário LISC, http://data.gbif.org/datasets/resource/12700  Millenium Seedbank (MSB), http://data.gbif.org/datasets/resource/12714  Missouri Botanical Garden, http://data.gbif.org/datasets/resource/12084  Nationaal Herbarium Nederland, http://data.gbif.org/datasets/resource/11520  Natural History Museum, Vienna - Herbarium W, http://data.gbif.org/datasets/resource/13042  NMNH Botany Collections, http://data.gbif.org/datasets/resource/1874  Peabody Paleobotany DiGIR Service, http://data.gbif.org/datasets/resource/8141  Peabody Paleoportal DiGIR Service (PB), http://data.gbif.org/datasets/resource/8176  Phanerogamic Botanical Collections (S), http://data.gbif.org/datasets/resource/8113  Phanerogams herbarium specimens, http://data.gbif.org/datasets/resource/13686  Precis Plant Data, http://data.gbif.org/datasets/resource/13505  PRECIS, http://data.gbif.org/datasets/resource/12716  Rapid Assessment Program (RAP) Biodiversity Survey Database, http://data.gbif.org/datasets/resource/8076  RBGE Herbarium (E), http://data.gbif.org/datasets/resource/8402  Real Jardin Botanico (Madrid), Vascular Plant Herbarium (MA), http://data.gbif.org/datasets/resource/240  Royal Botanic Gardens, Kew, http://data.gbif.org/datasets/resource/629  Royal Museum of Central Africa - Metafro-Infosys - Prelude, http://data.gbif.org/datasets/resource/96  Royal Museum of Central Africa - Metafro-Infosys - Xylarium, http://data.gbif.org/datasets/resource/14521  Southern Cape herbarium, http://data.gbif.org/datasets/resource/11954  Staatliches Museum für Naturkunde Stuttgart, Herbarium, http://data.gbif.org/datasets/resource/1100  SysTax - Botanical Gardens, http://data.gbif.org/datasets/resource/14438  The AAU Herbarium Database, http://data.gbif.org/datasets/resource/224  The Vascular Plant Collection at the Botanische Staatssammlung München, http://data.gbif.org/datasets/resource/11996  United States National Plant Germplasm System Collection, http://data.gbif.org/datasets/resource/1429  University of Ghana - Ghana Herbarium, http://data.gbif.org/datasets/resource/14195  West African Vegetation Database, http://data.gbif.org/datasets/resource/14161  West-, Central- and East African Plants Databases, http://data.gbif.org/datasets/resource/14190 |
| **Fabaceae** |  |
| ***Afzelia quanzensis* Welw.** | Base de datos para la xiloteca del Instituto de Biología de la UNAM, http://data.gbif.org/datasets/resource/13386  Botanic Garden of Finnish Museum of Natural History, http://data.gbif.org/datasets/resource/2406  Botany (UPS), http://data.gbif.org/datasets/resource/1045  EURISCO, The European Genetic Resources Search Catalogue, http://data.gbif.org/datasets/resource/1905  Herbarium Berolinense, http://data.gbif.org/datasets/resource/1095  Herbarium Senckenbergianum (FR), http://data.gbif.org/datasets/resource/8311  Herbarium Specimen of the Institute of Traditional Medicine, Tanzania, http://data.gbif.org/datasets/resource/13540  IICT Herbário LISC, http://data.gbif.org/datasets/resource/12700  Millenium Seedbank (MSB), http://data.gbif.org/datasets/resource/12714  Missouri Botanical Garden, http://data.gbif.org/datasets/resource/12084  Nationaal Herbarium Nederland, http://data.gbif.org/datasets/resource/11520  NMNH Botany Collections, http://data.gbif.org/datasets/resource/1874  Phanerogamic Botanical Collections (S), http://data.gbif.org/datasets/resource/8113  Phanerogams herbarium specimens, http://data.gbif.org/datasets/resource/13686  PRECIS (KwaZulu-Natal Herbarium), http://data.gbif.org/datasets/resource/12715  Precis Plant Data, http://data.gbif.org/datasets/resource/13505  PRECIS, http://data.gbif.org/datasets/resource/12716  RBGE Herbarium (E), http://data.gbif.org/datasets/resource/8402  Real Jardin Botanico (Madrid), Vascular Plant Herbarium (MA), http://data.gbif.org/datasets/resource/240  Royal Botanic Gardens, Kew, http://data.gbif.org/datasets/resource/629  Royal Museum of Central Africa - Metafro-Infosys - Prelude, http://data.gbif.org/datasets/resource/96  SysTax - Botanical Gardens, http://data.gbif.org/datasets/resource/14438  United States National Plant Germplasm System Collection, http://data.gbif.org/datasets/resource/1429  West-, Central- and East African Plants Databases, http://data.gbif.org/datasets/resource/14190 |
| ***Gossweilerodendron balsamiferum* (Vermoesen) Harms** | Computarización de la xiloteca Dr. Faustino Miranda del Instituto de Ecología, AC, http://data.gbif.org/datasets/resource/13104  IICT Herbário LISC, http://data.gbif.org/datasets/resource/12700  Missouri Botanical Garden, http://data.gbif.org/datasets/resource/12084  Nationaal Herbarium Nederland, http://data.gbif.org/datasets/resource/11520  NMNH Botany Collections, http://data.gbif.org/datasets/resource/1874  Phanerogams herbarium specimens, http://data.gbif.org/datasets/resource/13686  Rapid Assessment Program (RAP) Biodiversity Survey Database, http://data.gbif.org/datasets/resource/8076  Real Jardin Botanico (Madrid), Vascular Plant Herbarium (MA), http://data.gbif.org/datasets/resource/240  Royal Botanic Gardens, Kew, http://data.gbif.org/datasets/resource/629 |
| ***Guibourtia arnoldiana* (De Wild. & T. Durand) J. Léonard** | Computarización de la xiloteca Dr. Faustino Miranda del Instituto de Ecología, AC, http://data.gbif.org/datasets/resource/13104  IICT Herbário LISC, http://data.gbif.org/datasets/resource/12700  Missouri Botanical Garden, http://data.gbif.org/datasets/resource/12084  Nationaal Herbarium Nederland, http://data.gbif.org/datasets/resource/11520  NMNH Botany Collections, http://data.gbif.org/datasets/resource/1874  Phanerogams herbarium specimens, http://data.gbif.org/datasets/resource/13686  Rapid Assessment Program (RAP) Biodiversity Survey Database, http://data.gbif.org/datasets/resource/8076  Real Jardin Botanico (Madrid), Vascular Plant Herbarium (MA), http://data.gbif.org/datasets/resource/240  Royal Botanic Gardens, Kew, http://data.gbif.org/datasets/resource/629 |
| ***Guibourtia coleosperma* (Benth.) J. Léonard** | EURISCO, The European Genetic Resources Search Catalogue, http://data.gbif.org/datasets/resource/1905  Herbarium Berolinense, http://data.gbif.org/datasets/resource/1095  IICT Herbário LISC, http://data.gbif.org/datasets/resource/12700  Missouri Botanical Garden, http://data.gbif.org/datasets/resource/12084  Nationaal Herbarium Nederland, http://data.gbif.org/datasets/resource/11520  NMNH Botany Collections, http://data.gbif.org/datasets/resource/1874  Phanerogams herbarium specimens, http://data.gbif.org/datasets/resource/13686  Precis Plant Data, http://data.gbif.org/datasets/resource/13505  PRECIS, http://data.gbif.org/datasets/resource/12716  Royal Botanic Gardens, Kew, http://data.gbif.org/datasets/resource/629  Royal Museum of Central Africa - Metafro-Infosys - Prelude, http://data.gbif.org/datasets/resource/96  Southern Cape herbarium, http://data.gbif.org/datasets/resource/11954  SysTax - Botanical Gardens, http://data.gbif.org/datasets/resource/14438  UA Herbarium, http://data.gbif.org/datasets/resource/7900  United States National Plant Germplasm System Collection, http://data.gbif.org/datasets/resource/1429 |
| ***Oxystigma oxyphyllum* (Harms) J. Léonard** | Cameroon National Herbarium, http://data.gbif.org/datasets/resource/1474  IICT Herbário LISC, http://data.gbif.org/datasets/resource/12700  Missouri Botanical Garden, http://data.gbif.org/datasets/resource/12084  Nationaal Herbarium Nederland, http://data.gbif.org/datasets/resource/11520  NMNH Botany Collections, http://data.gbif.org/datasets/resource/1874  Phanerogams herbarium specimens, http://data.gbif.org/datasets/resource/13686  PRECIS, http://data.gbif.org/datasets/resource/12716  Rapid Assessment Program (RAP) Biodiversity Survey Database, http://data.gbif.org/datasets/resource/8076 |
| ***Pterocarpus angolensis* DC.** | Base de datos para la xiloteca del Instituto de Biología de la UNAM, http://data.gbif.org/datasets/resource/13386  EURISCO, The European Genetic Resources Search Catalogue, http://data.gbif.org/datasets/resource/1905  Herbarium Berolinense, http://data.gbif.org/datasets/resource/1095  Herbarium Specimen of the Institute of Traditional Medicine, Tanzania, http://data.gbif.org/datasets/resource/13540  IICT Herbário LISC, http://data.gbif.org/datasets/resource/12700  Missouri Botanical Garden, http://data.gbif.org/datasets/resource/12084  Nationaal Herbarium Nederland, http://data.gbif.org/datasets/resource/11520  NMNH Botany Collections, http://data.gbif.org/datasets/resource/1874  Phanerogamic Botanical Collections (S), http://data.gbif.org/datasets/resource/8113  Phanerogams herbarium specimens, http://data.gbif.org/datasets/resource/13686  Precis Plant Data, http://data.gbif.org/datasets/resource/13505  PRECIS, http://data.gbif.org/datasets/resource/12716  RBGE Herbarium (E), http://data.gbif.org/datasets/resource/8402  Real Jardin Botanico (Madrid), Vascular Plant Herbarium (MA), http://data.gbif.org/datasets/resource/240  Royal Botanic Gardens, Kew, http://data.gbif.org/datasets/resource/629  Royal Museum of Central Africa - Metafro-Infosys - Prelude, http://data.gbif.org/datasets/resource/96  SINGER Coordinator, http://data.gbif.org/datasets/resource/8349  The System-wide Information Network for Genetic Resources (SINGER), http://data.gbif.org/datasets/resource/1430  United States National Plant Germplasm System Collection, http://data.gbif.org/datasets/resource/1429  University of Ghana - Ghana Herbarium, http://data.gbif.org/datasets/resource/14195  West African Vegetation Database, http://data.gbif.org/datasets/resource/14161  West-, Central- and East African Plants Databases, http://data.gbif.org/datasets/resource/14190 |
| ***Pterocarpus tinctorius* Welw** | Botany (UPS), http://data.gbif.org/datasets/resource/1045  EURISCO, The European Genetic Resources Search Catalogue, http://data.gbif.org/datasets/resource/1905  Harvard University Herbaria, http://data.gbif.org/datasets/resource/1827  Herbaria of the University and ETH Zürich, http://data.gbif.org/datasets/resource/11516  Herbarium of The New York Botanical Garden, http://data.gbif.org/datasets/resource/8967  Herbarium Specimen of the Institute of Traditional Medicine, Tanzania, http://data.gbif.org/datasets/resource/13540  IICT Herbário LISC, http://data.gbif.org/datasets/resource/12700  Missouri Botanical Garden, http://data.gbif.org/datasets/resource/12084  Nationaal Herbarium Nederland, http://data.gbif.org/datasets/resource/11520  NMNH Botany Collections, http://data.gbif.org/datasets/resource/1874  Phanerogamic Botanical Collections (S), http://data.gbif.org/datasets/resource/8113  Phanerogams herbarium specimens, http://data.gbif.org/datasets/resource/13686  RBGE Herbarium (E), http://data.gbif.org/datasets/resource/8402  Real Jardin Botanico (Madrid), Vascular Plant Herbarium (MA), http://data.gbif.org/datasets/resource/240  Royal Botanic Gardens, Kew, http://data.gbif.org/datasets/resource/629 |
| ***Bobgunnia fistuloides* (Harms) J.H. Kirkbr. & Wiersema** | Harvard University Herbaria, http://data.gbif.org/datasets/resource/1827  Herbarium Berolinense, http://data.gbif.org/datasets/resource/1095  IICT Herbário LISC, http://data.gbif.org/datasets/resource/12700  Missouri Botanical Garden, http://data.gbif.org/datasets/resource/12084  Nationaal Herbarium Nederland, http://data.gbif.org/datasets/resource/11520  Natural History Museum, Vienna - Herbarium W, http://data.gbif.org/datasets/resource/13042  NMNH Botany Collections, http://data.gbif.org/datasets/resource/1874  Phanerogams herbarium specimens, http://data.gbif.org/datasets/resource/13686  PRECIS, http://data.gbif.org/datasets/resource/12716  RBGE Herbarium (E), http://data.gbif.org/datasets/resource/8402  Real Jardin Botanico (Madrid), Vascular Plant Herbarium (MA), http://data.gbif.org/datasets/resource/240  Royal Botanic Gardens, Kew, http://data.gbif.org/datasets/resource/629  University of Ghana - Ghana Herbarium, http://data.gbif.org/datasets/resource/14195 |
| **Meliaceae** |  |
| ***Entandrophragma angolense* (Welw.) C. DC.** | Botanical Museum, Copenhagen. Database of type specimens, http://data.gbif.org/datasets/resource/716  Field Museum of Natural History (Botany) Seed Plant Collection, http://data.gbif.org/datasets/resource/14346  Herbarium of The New York Botanical Garden, http://data.gbif.org/datasets/resource/8967  IICT Herbário LISC, http://data.gbif.org/datasets/resource/12700  Missouri Botanical Garden, http://data.gbif.org/datasets/resource/12084  Nationaal Herbarium Nederland, http://data.gbif.org/datasets/resource/11520  NMNH Botany Collections, http://data.gbif.org/datasets/resource/1874  Peabody Paleobotany DiGIR Service, http://data.gbif.org/datasets/resource/8141  Peabody Paleoportal DiGIR Service (PB), http://data.gbif.org/datasets/resource/8176  Phanérogames recensés aux Monts Nimba, http://data.gbif.org/datasets/resource/13405  Phanerogams herbarium specimens, http://data.gbif.org/datasets/resource/13686  Precis Plant Data, http://data.gbif.org/datasets/resource/13505  PRECIS, http://data.gbif.org/datasets/resource/12716  Rapid Assessment Program (RAP) Biodiversity Survey Database, http://data.gbif.org/datasets/resource/8076  RBGE Herbarium (E), http://data.gbif.org/datasets/resource/8402  RBGE Living Collections, http://data.gbif.org/datasets/resource/9167  Real Jardin Botanico (Madrid), Vascular Plant Herbarium (MA), http://data.gbif.org/datasets/resource/240  Royal Botanic Gardens, Kew, http://data.gbif.org/datasets/resource/629  Royal Museum of Central Africa - Metafro-Infosys - Xylarium, http://data.gbif.org/datasets/resource/14521  The Vascular Plant Collection at the Botanische Staatssammlung München, http://data.gbif.org/datasets/resource/11996  University of Ghana - Ghana Herbarium, http://data.gbif.org/datasets/resource/14195 |
| ***Entandrophragma candollei* Harms** | Base de datos para la xiloteca del Instituto de Biología de la UNAM, http://data.gbif.org/datasets/resource/13386  Herbarium of the Université Libre de Bruxelles, http://data.gbif.org/datasets/resource/9102  IICT Herbário LISC, http://data.gbif.org/datasets/resource/12700  Missouri Botanical Garden, http://data.gbif.org/datasets/resource/12084  Nationaal Herbarium Nederland, http://data.gbif.org/datasets/resource/11520  NMNH Botany Collections, http://data.gbif.org/datasets/resource/1874  Peabody Paleobotany DiGIR Service, http://data.gbif.org/datasets/resource/8141  Peabody Paleoportal DiGIR Service (PB), http://data.gbif.org/datasets/resource/8176  Phanérogames recensés aux Monts Nimba, http://data.gbif.org/datasets/resource/13405  Phanerogams herbarium specimens, http://data.gbif.org/datasets/resource/13686  Rapid Assessment Program (RAP) Biodiversity Survey Database, http://data.gbif.org/datasets/resource/8076  Real Jardin Botanico (Madrid), Vascular Plant Herbarium (MA), http://data.gbif.org/datasets/resource/240  Royal Botanic Gardens, Kew, http://data.gbif.org/datasets/resource/629  Royal Museum of Central Africa - Metafro-Infosys - Xylarium, http://data.gbif.org/datasets/resource/14521  University of Ghana - Ghana Herbarium, http://data.gbif.org/datasets/resource/14195 |
| ***Entandrophragma cylindricum* (Sprague) Sprague** | Herbarium of The New York Botanical Garden, http://data.gbif.org/datasets/resource/8967  Herbarium togoense, http://data.gbif.org/datasets/resource/12767  IICT Herbário LISC, http://data.gbif.org/datasets/resource/12700  Missouri Botanical Garden, http://data.gbif.org/datasets/resource/12084  Nationaal Herbarium Nederland, http://data.gbif.org/datasets/resource/11520  NMNH Botany Collections, http://data.gbif.org/datasets/resource/1874  Phanérogames recensés aux Monts Nimba, http://data.gbif.org/datasets/resource/13405  Phanerogams herbarium specimens, http://data.gbif.org/datasets/resource/13686  Rapid Assessment Program (RAP) Biodiversity Survey Database, http://data.gbif.org/datasets/resource/8076  Real Jardin Botanico (Madrid), Vascular Plant Herbarium (MA), http://data.gbif.org/datasets/resource/240  Royal Botanic Gardens, Kew, http://data.gbif.org/datasets/resource/629  Royal Museum of Central Africa - Metafro-Infosys - Xylarium, http://data.gbif.org/datasets/resource/14521  University of Ghana - Ghana Herbarium, http://data.gbif.org/datasets/resource/14195 |
| ***Entandrophragma spicatum* (C. DC.) Sprague** | HBGSpermatophyta - Herbarium Hamburgense, http://data.gbif.org/datasets/resource/1604  IICT Herbário LISC, http://data.gbif.org/datasets/resource/12700  Precis Plant Data, http://data.gbif.org/datasets/resource/13505  PRECIS, http://data.gbif.org/datasets/resource/12716  Royal Botanic Gardens, Kew, http://data.gbif.org/datasets/resource/629  Royal Museum of Central Africa - Metafro-Infosys - Xylarium, http://data.gbif.org/datasets/resource/14521  The Vascular Plant Collection at the Botanische Staatssammlung München, http://data.gbif.org/datasets/resource/11996 |
| ***Entandrophragma utile* (Dauwe & Sprague) Sprague** | Herbarium of the Université Libre de Bruxelles, http://data.gbif.org/datasets/resource/9102  IICT Herbário LISC, http://data.gbif.org/datasets/resource/12700  Missouri Botanical Garden, http://data.gbif.org/datasets/resource/12084  Nationaal Herbarium Nederland, http://data.gbif.org/datasets/resource/11520  NMNH Botany Collections, http://data.gbif.org/datasets/resource/1874  Phanérogames recensés aux Monts Nimba, http://data.gbif.org/datasets/resource/13405  Phanerogams herbarium specimens, http://data.gbif.org/datasets/resource/13686  Rapid Assessment Program (RAP) Biodiversity Survey Database, http://data.gbif.org/datasets/resource/8076  RBGE Living Collections, http://data.gbif.org/datasets/resource/9167  Real Jardin Botanico (Madrid), Vascular Plant Herbarium (MA), http://data.gbif.org/datasets/resource/240  Royal Botanic Gardens, Kew, http://data.gbif.org/datasets/resource/629  Royal Museum of Central Africa - Metafro-Infosys - Xylarium, http://data.gbif.org/datasets/resource/14521  University of Ghana - Ghana Herbarium, http://data.gbif.org/datasets/resource/14195 |
| ***Khaya anthotheca* (Welw.) C. DC.** | Botanic Garden of Finnish Museum of Natural History, http://data.gbif.org/datasets/resource/2406  Botany (UPS), http://data.gbif.org/datasets/resource/1045  CONN, http://data.gbif.org/datasets/resource/13544  Fairchild Tropical Botanic Garden Virtual Herbarium Darwin Core format, http://data.gbif.org/datasets/resource/202  Field Museum of Natural History (Botany) Seed Plant Collection, http://data.gbif.org/datasets/resource/14346  Harvard University Herbaria, http://data.gbif.org/datasets/resource/1827  Herbarium Berolinense, http://data.gbif.org/datasets/resource/1095  Herbarium of The New York Botanical Garden, http://data.gbif.org/datasets/resource/8967  Herbarium Specimen of the Institute of Traditional Medicine, Tanzania, http://data.gbif.org/datasets/resource/13540  IICT Herbário LISC, http://data.gbif.org/datasets/resource/12700  Missouri Botanical Garden, http://data.gbif.org/datasets/resource/12084  Nationaal Herbarium Nederland, http://data.gbif.org/datasets/resource/11520  NMNH Botany Collections, http://data.gbif.org/datasets/resource/1874  Peabody Paleobotany DiGIR Service, http://data.gbif.org/datasets/resource/8141  Peabody Paleoportal DiGIR Service (PB), http://data.gbif.org/datasets/resource/8176  Phanerogamic Botanical Collections (S), http://data.gbif.org/datasets/resource/8113  Phanerogams herbarium specimens, http://data.gbif.org/datasets/resource/13686  Rapid Assessment Program (RAP) Biodiversity Survey Database, http://data.gbif.org/datasets/resource/8076  RBGE Herbarium (E), http://data.gbif.org/datasets/resource/8402  RBGE Living Collections, http://data.gbif.org/datasets/resource/9167  Real Jardin Botanico (Madrid), Vascular Plant Herbarium (MA), http://data.gbif.org/datasets/resource/240  Royal Botanic Gardens, Kew, http://data.gbif.org/datasets/resource/629  Royal Museum of Central Africa - Metafro-Infosys - Prelude, http://data.gbif.org/datasets/resource/96  Royal Museum of Central Africa - Metafro-Infosys - Xylarium, http://data.gbif.org/datasets/resource/14521  SysTax - Botanical Gardens, http://data.gbif.org/datasets/resource/14438  The Vascular Plant Collection at the Botanische Staatssammlung München, http://data.gbif.org/datasets/resource/11996  University of Copenhagen's Arboretum, http://data.gbif.org/datasets/resource/702  University of Ghana - Ghana Herbarium, http://data.gbif.org/datasets/resource/14195  USDA PLANTS Database, http://data.gbif.org/datasets/resource/1066  WAHerb, http://data.gbif.org/datasets/resource/13675  West-, Central- and East African Plants Databases, http://data.gbif.org/datasets/resource/14190 |
| ***Khaya ivorensis* A. Chev.** | Computarización de la xiloteca Dr. Faustino Miranda del Instituto de Ecología, AC, http://data.gbif.org/datasets/resource/13104  Herbarium Berolinense, http://data.gbif.org/datasets/resource/1095  Herbarium of the Université Libre de Bruxelles, http://data.gbif.org/datasets/resource/9102  IICT Herbário LISC, http://data.gbif.org/datasets/resource/12700  Missouri Botanical Garden, http://data.gbif.org/datasets/resource/12084  Nationaal Herbarium Nederland, http://data.gbif.org/datasets/resource/11520  NMNH Botany Collections, http://data.gbif.org/datasets/resource/1874  Phanérogames recensés aux Monts Nimba, http://data.gbif.org/datasets/resource/13405  Phanerogams herbarium specimens, http://data.gbif.org/datasets/resource/13686  Rapid Assessment Program (RAP) Biodiversity Survey Database, http://data.gbif.org/datasets/resource/8076  RBGE Herbarium (E), http://data.gbif.org/datasets/resource/8402  RBGE Living Collections, http://data.gbif.org/datasets/resource/9167  Real Jardin Botanico (Madrid), Vascular Plant Herbarium (MA), http://data.gbif.org/datasets/resource/240  Royal Museum of Central Africa - Metafro-Infosys - Xylarium, http://data.gbif.org/datasets/resource/14521  University of Ghana - Ghana Herbarium, http://data.gbif.org/datasets/resource/14195 |
| **Moraceae** |  |
| ***Milicia excelsa* (Welw.) C.C. Berg** | Base de datos para la xiloteca del Instituto de Biología de la UNAM, http://data.gbif.org/datasets/resource/13386  Bishop Museum Natural Sciences Data, http://data.gbif.org/datasets/resource/54  Botany (UPS), http://data.gbif.org/datasets/resource/1045  Cameroon National Herbarium, http://data.gbif.org/datasets/resource/1474  EURISCO, The European Genetic Resources Search Catalogue, http://data.gbif.org/datasets/resource/1905  Harvard University Herbaria, http://data.gbif.org/datasets/resource/1827  Herbarium Berolinense, http://data.gbif.org/datasets/resource/1095  Herbarium of the Université Libre de Bruxelles, http://data.gbif.org/datasets/resource/9102  Herbarium Senckenbergianum (FR), http://data.gbif.org/datasets/resource/8311  Herbarium togoense, http://data.gbif.org/datasets/resource/12767  Herbarium WU, http://data.gbif.org/datasets/resource/1496  Herbier du Bénin, http://data.gbif.org/datasets/resource/13459  IICT Herbário LISC, http://data.gbif.org/datasets/resource/12700  Jardín Botánico de Córdoba: Herbarium COA, http://data.gbif.org/datasets/resource/247  Missouri Botanical Garden, http://data.gbif.org/datasets/resource/12084  Nationaal Herbarium Nederland, http://data.gbif.org/datasets/resource/11520  NMNH Botany Collections, http://data.gbif.org/datasets/resource/1874  Phanérogames recensés aux Monts Nimba, http://data.gbif.org/datasets/resource/13405  Phanerogamic Botanical Collections (S), http://data.gbif.org/datasets/resource/8113  Phanerogams herbarium specimens, http://data.gbif.org/datasets/resource/13686  PRECIS, http://data.gbif.org/datasets/resource/12716  Rapid Assessment Program (RAP) Biodiversity Survey Database, http://data.gbif.org/datasets/resource/8076  RBGE Herbarium (E), http://data.gbif.org/datasets/resource/8402  Real Jardin Botanico (Madrid), Vascular Plant Herbarium (MA), http://data.gbif.org/datasets/resource/240  Royal Botanic Gardens, Kew, http://data.gbif.org/datasets/resource/629  Royal Museum of Central Africa - Metafro-Infosys - Xylarium, http://data.gbif.org/datasets/resource/14521  SysTax - Botanical Gardens, http://data.gbif.org/datasets/resource/14438  United States National Plant Germplasm System Collection, http://data.gbif.org/datasets/resource/1429  University of Ghana - Ghana Herbarium, http://data.gbif.org/datasets/resource/14195  West-, Central- and East African Plants Databases, http://data.gbif.org/datasets/resource/14190 |
