# Supplementary material for: Documenting Biogeographical Patterns of African Timber Species Using Herbarium Records: A Conservation Perspective Based on Native Trees from Angola
Source: PLoS One. 2014 Jul 25;9(7):e103403. doi: 10.1371/journal.pone.0103403 (PMC4111583; doi:10.1371/journal.pone.0103403)
Supplement: Table S4 — Characteristics of the 18 timber species studied, including information about their family, synonymy, common names, habit and ecology, and timber characteristics and uses. (DOC) [file pone.0103403.s006.doc]

**Table S4.** Characteristics of the 18 timber species studied, including information about their family, synonymy, common names, habit and ecology, and timber characteristics and uses.

| **Family, species and synonyms** | | **Common names** | **Habit and ecology*** | **Timber characteristics and uses*** |
| --- | --- | --- | --- | --- |
| **Combretaceae** | |  |  |  |
|  | ***Terminalia superba* Engl. & Diels** | limba (trade name); mulimba (Kikongo): Cabinda, Maiombe. | Tree, up to 50 m high. Along riverbanks and in lagoons that is flooded during 1-2 months year. | In Angola “Limba” is restricted to Cabinda and has a high commercial value, being one of the market’s favourite timbers. It is easy to work, producing a good finish. It is used in veneer, plywood, carpentry, frames, various constructions, railway sleepers, etc. |
| **Ebenaceae** | |  |  |  |
|  | ***Diospyros mespiliformis* Hochst. ex A. DC.** | musolveira (portuguese): Cuanza-Norte; mulende (Kimbundu); unnhandi (Lunyaneka): Huíla, Gambos; omuandi (Xikuanhama): Baixo Cunene, Ondjiva; omuandi, (Lunyaneka): Serra Chela; icolo; African ebony (trade name). | Tree or shrub, up to 20m high. It frequently grows on stony soils, along riverbanks and water courses, in open woodlands. | This species produces high quality ebony. This wood is light-colored or slightly reddish, darkening almost to black after cutting and exposure to air. The wood is long-lasting and easy to work, suitable for construction and furniture, fine carpentry, plywoods, tool handles, woodturning, etc. |
| **Fabaceae** | |  |  |  |
|  | ***Afzelia quanzensis* Welw.** | ovala, uvala, muvala, muala-to (Ganguela); mupazu, pupopo (Kioko); omulatobo, muvanje (Umbundu); omubantya (Tjiherero); mukungu, mbange (Kimbundu); omuanki (Lunyaneka); chanfuta (trade name). | Tree or shrub, up to 20m high. In dry forests and savannas, growing in isolation or in small populations. | The wood is brown-yellowish, becoming brown-reddish after exposure to light, similarly to mahogany; it is hard and rarely attacked by insects and fungi. The wood is used in carpentry, woodwork, construction and shipbuilding. |
|  | ***Gossweilerodendron balsamiferum* (Vermoesen) Harms**  *=*[*Prioria balsamifera* (Vermoesen) Breteler](http://www.ville-ge.ch/musinfo/bd/cjb/africa/details.php?langue=an&id=71103)  *=Pterygopodium balsamiferum* Vermoesen | tola branca (nome colonial); kitola (Kikongo): Cabinda; mupéle (Kikongo): Maiombe-Zaire; tola; quitola; ktola; moboron; mutala-menha (Quimbundo); mafuta; agba (trade name) | Tree, up to 40m high. Abundant in Lower Maiombe. In dense moist forests, in isolated stands. | The wood is slightly pinkish and light-coloured, with a pattern similar to that of mahogany. It is used to replace conifer timbers because it is light and has several applications. Is used in shipbuilding and building construction, carpentry, railway sleepers, etc. |
|  | ***Guibourtia arnoldiana* (De Wild. & T. Durand) J. Léonard**  *=Copaiba arnoldiana* De Wild. & T. Durand;  =*Copaifera arnoldiana* (De Wild. & T. Durand) T. Durand & H. Durand | benje; penje ou mpenze (Kikongo); mutenye (trade name) | Tree, up to 30m high. In dense moist forests. | The wood consists of white sapwood and brown heartwood, and is very hard and heavy; it is very strong and easy to work. It is much appreciated for use in furniture, carpentry and woodwork, parquets. Due to its strength, it is suitable for constructing trucks, wagon interiors, shipbuilding, railway sleepers, etc. |
|  | ***Guibourtia coleosperma* (Benth.) J. Léonard**  *=Copaifera coleosperma* Benth. | mussibi; mucive; musci; mushii; misivi e muxivi (Ganguela); muxi (Kioko); muxibi (Umbundu); omusivi (Xikuanhama); African rosewood (trade name). | Tree, up to 25m high. In tropical dry forest, being one of the tallest trees found between Lower Cubango. | This species’ wood is quite similar to *Pterocarpus tinctorius*, being an attractive striped red; it is very durable and easily cut. Used for railway sleepers, woodwork, shipbuilding, parquet blocks, plywoods, and industrial vessels and containers. It is also used by local craftsmen to produce valuable art pieces. |
|  | ***Oxystigma oxyphyllum* (Harms) J. Léonard**  *=Prioria oxyphylla* (Harms) Breteler  *=Pterygopodium oxyphyllum* Harms | tola chinfuta; banda; t´chinfuta; kitola-kinfuta (Kikongo): Cabinda; tchitola (trade name) | Tree, up to 30m high. In dense moist forests, growing in isolation or in small populations. | It has good characteristics being highly durable, strong and easy to work, with qualities comparable to certain African mahoganies. This wood is primarily used for shipbuilding and building construction, woodwork and decorative work, wooden boxes and packages, plywoods and veneers. |
|  | ***Pterocarpus angolensis* DC.**  *=Pterocarpus erinaceus* sensu Baker | girassonde; mucula; mupoamalengue; mu´huva; murilahonde; mirahonde; njila-sonde, mutete, mudilahonde ou kaionga (Kimbundo); milahondi, omuliahonde, omuliahonde (Lunyaneka); njilasonde ou mukula (Ganguela); muva ou omuva (Xikuanhama); muninga (trade name). | Tree, up to 20m high. In open forest and tree savanna. | The wood is darkish-brown and reddish, easy to work and allowing a good finish. The timber is described as quite resistant and excellent for building construction boats, veneers, plywoods and pavements. Due to its high durability, it is often used in works exposed to the elements. It is also suitable for medicinal applications; for example, the resin - locally called “dragon blood” or “kino” - is used to heal wounds. |
|  | ***Pterocarpus tinctorius* Welw**  *=Pterocarpus cabrae* sensu Baker f. | tacula, takula, hula or muange (Kimbundu); lukunga (Kikongo); lucula; cuula; padouk (trade name) | Tree, up to 20m high. Along riverbanks; it also frequently grows in the dense moist forest of the Dembos and North-Cuanza regions | The wood is reddish or whitish and it is easy to work. Due to its characteristics, it is much sought after for building construction and shipbuilding, woodwork, carpentry and decoration. This wood can also be used to produce a fine powder which is mixed with oil to prepare red inks, used for various purposes. |
|  | ***Bobgunnia fistuloides* (Harms) J.H. Kirkbr. & Wiersema**  *=Swartzia fistuloides* Harms | pau rosa; sambu (Kikongo); sassambu; sambo-sambo; dina (trade name) | Tree, medium to large-sized, height 25m or more. In dense moist forest and near running water. | Wood has a fine, quite interlocked grain. The light-brown sapwood is easily distinguishable from the brown-pink heartwood, with longitudinal darker patches, sometimes reddish or violet. It produces fine decorative pieces, woodwork and turned pieces; smaller boards are used for trays, boxes and small objects; it is also used for veneers, railway sleepers, etc. |
| **Meliaceae** | |  |  |  |
|  | ***Entandrophragma angolense* (Welw.) C. DC.**  *=Swietenia angolensis* Welw.  =*Entandrophragma septentrionale* A. Chev. | African mahogany; njimba-muxi, or ngimbe-muxi (Kimbundu): Cazengo; vialunji, or kalunji (Kikongo): Maiombe, Buco Zau; kibemuxi (Kimbundu): Cuanza-Norte; mupapala (Kioko): Lunda; susua (Umbundu): Cuanza-Sul, Amboim; kitiva (Kimbundu): Dembos; tiana; gedu nohor (trade name). | Tree, to 40m high or more. In moist, dense forests. | The tree has distinct, lighter-colored sapwood; coarse grained, soft and moderately heavy; easily sawn. The wood is very decorative and used in plywood, joinery, carpentry and veneers. |
|  | ***Entandrophragma candollei* Harms** | African mahogany, likufi; undianunu (Kikongo): Maiombe, Buco Zau; kosipo.kosipo (trade name) | Tree, up to 50m high. In dense rainforests. | This tree species is much exploited for the excellent quality of its mahogany. The wood is particularly sought after for plywood and joinery. |
|  | ***Entandrophragma cylindricum* (Sprague) Sprague** | African mahogany; livuite; lifuti, linvuti, or lifumo (Kikongo): Cabinda, Buco Zau, Maiombe; undianuno de casca fina; monguba; sapelli; sapele (trade name) | Tree, up to 35m high. In dense rainforests. | The wood is pinkish upon cutting; it then becomes reddish-brown; very fine-grained. Particularly sought after for plywood, veneer, joinery, decoration and building construction. |
|  | ***Entandrophragma spicatum* (C. DC.) Sprague**  *=Wulfhorstia spicata* C. DC.  *=Wulfhorstia ekebergioides* Harms  *=Entandrophragma ekebergioides* (Harms) Sprague | African mahogany; omumbili (Lunyaneka): Serra de Chela, Huíla; omutaku, (Lunyaneka): Huíla, Moçâmedes; mahoni; omutaku. | Tree, up to 20m high. In dry tropical savannas, and between stones in rocky areas. | The brown-coloured wood is the heaviest and hardest of all Angolan mahoganies; its board does not warp, but it fractures easily if not properly handled. This is one of the most remarkable tropical dry forest species, highly valued for woodwork and handiwork. |
|  | ***Entandrophragma utile* (Dauwe & Sprague) Sprague** | African mahogany; lifuti; kalunji; undianunu (Kikongo): Maiombe, Buco Zau; sipo; utile (trade name). | Tree, 40 to 50m high. In dense moist forest. | This tree species produces a pinkish-brown mahogany. This wood is quite heavy and strong and easy to work. It is also used in plywoods, joinery, carpentry and veneers. |
|  | ***Khaya anthotheca* (Welw.) C. DC.**  *=Garretia anthotheca* Welw. | African mahogany; quibaba; kibaba de mussengue; muhumbu (Kikongo): Congo, Uíge; n´dola: Zaire; acajou de Africa (international), acajou blanc. | Tree, generally to 30m high. In the coffee-producing regions of South-Cuanza, North-Cuanza and in some forests in Malange. | The wood is reddish-brown and easy to work, durable, and resistant to fungi and insects. It has the same applications as other mahoganies: joinery, carpentry, building construction and shipbuilding, veneers, plywoods, train wagons, etc. The bark is used in Angola to fight certain diseases because it has remarkable and energetic antipyretic properties. |
|  | ***Khaya ivorensis* A. Chev.**  *=Khaya klainei* Pierre ex Pellegr. | African mahogany; undianunu, or lituti (Kikongo): Cabinda, Maiombe; acajou de Africa. | Tree, height normally 25m, sometimes higher. In dense rainforests of the Maiombe, along the Luango river. | The brown wood is a true mahogany. It is sought after mainly for plywoods, fine joinery, veneers, decorations, small boats and especially furniture. |
| **Moraceae** | |  |  |  |
|  | ***Milicia excelsa* (Welw.) C.C. Berg**  *=Morus excelsa* Welw.  *=Chlorophora excelsa* (Welw.) Benth. & Hook.f. | amoreira or moreira (Portuguese); mukuma, or mukamba-kamba (Kimbundu): Cuanza-Norte, Welw.; mova (Kioko): Moxico, Luau; sanga: Lunda, Dundo; makamba (Kioko): Moxico, Luau; kamba (Umbundu): Cuanza-Sul, Amboim; kambula (Kikongo): Cabinda, Maiombe; cambala; iroko (trade name). | Tree, 40 to 60m high. In moist forests; sometimes can be found in savannas, where its height does not exceed 30m. | This species has yellowish-brown fresh wood, which progressively changes to dark brown after sawing; distinct heartwood and sapwood; good finishing, especially after polishing. This wood is sought after for these characteristics, mainly for building construction and shipbuilding, joinery and carpentry, furniture, decoration, plywoods, industrial vessels and containers. |

*Information based on the study of herbarium specimens and bibliography [28, 29, 30, 31, 32, 36, 37, 38, 39, 40, 41, 42, 43, 44].
